# Framing reproductive narratives: A thematic discourse analysis of news representations of childlessness in 86 countries (2015–2025)

**DOI:** 10.1371/journal.pgph.0005695

**Published:** 2026-03-11

**Authors:** Sitta Fiakhsani Taqwim, Wenqian Xu, Yi Hyun Kang, Huzeifa Aweesha, Rashmi Rashmi, Paul Joseph Amani, Venosa Mushi, Rockli Kim, Julia Schröders

**Affiliations:** 1 Department of Epidemiology and Global Health, Umeå University, Umeå, Sweden; 2 Department of Health Sciences, Lund University, Lund, Sweden; 3 Department of Social Welfare, Seoul National University, Seoul, South Korea; 4 Department of Political Science, Lund University, Lund, Sweden; 5 International Institute of Population Sciences, Mumbai, India; 6 Department of Health System Management, Mzumbe University, Mzumbe, Tanzania; 7 Institute of Development Studies, Mzumbe University, Mzumbe, Tanzania; 8 Division of Health Policy and Management, Korea University, Seoul, South Korea; 9 Centre for Demographic and Ageing Research, Umeå University, Umeå, Sweden; Centre of Biomedical Ethics and Culture, PAKISTAN

## Abstract

Childlessness is an increasingly visible phenomenon. Once predominantly associated with high-income settings, it now spans diverse cultural, economic, and political contexts, including the Global South. Among recent demographic shifts, childlessness has emerged as one of the most ideologically charged and widely debated topics in public discourse, particularly through media narratives. Although media are often overlooked in mainstream public health models, they play critical roles as structural and intermediary determinants of health - shaping issue framing, amplifying voices, and legitimizing solutions. Yet little is known about how childlessness is represented in global media, especially outside the Global North and in the post-pandemic era. This study analysed news media representations of childlessness from a public health perspective, drawing on 131 news articles from 101 outlets across 86 countries (2015–2025). Articles were identified through systematic keyword searches in English and 12 additional languages, screened for relevance, and analysed thematically using Braun and Clarke’s inductive method. Our approach was discourse-sensitive, drawing on a social constructionist lens and informed by framing theories and reproductive justice. Five themes were identified: *The guinea pig of the state*; *Crazy rich selfish animal lovers*; *No baby, no cry*; *Bringing children into a broken world*; and *Winter regret and loneliness*. These narratives operate across structural, intermediary, and individual levels, fulfilling four discursive functions: politicising, moralising, pathologising, and humanising. By examining how childlessness is problematized or legitimized, this study highlights the media’s role in shaping reproductive narratives, stigma, and health equity across diverse contexts.

## Introduction

### Childlessness in a changing world

Childlessness – defined as the absence of biological, fostered or adopted children in an individual’s life, whether voluntary or involuntary [1] – is a socially complex phenomenon that has historically been framed as problematic, stigmatized, and misunderstood. While some experience childlessness as a result of biological, relational, or structural barriers, others deliberately choose not to become parents. The literature reflects this duality, debating whether childlessness is primarily a consequence of fertility postponement or a conscious life choice to reject parenthood. More recently the term “childless” has emerged as value-laden and deficit-oriented - for implying a lack - due to the suffix “less” – while the term “childfree” reflects empowerment, autonomy, and agency in reproductive decisions [2]. This indicates diverse experiences and circumstances of childless individuals, highlighting the need for a more nuanced understanding of this demographic group.

Globally, rates of childlessness are rising [3]. The trend is most pronounced in high income countries where the mean at marriage is high and entry into parenthood is on average more delayed, such as Austria, Germany, Italy, and the UK. The prevalence is particularly high in countries with widespread individualist values such as Finland or the Netherlands [4]. However, childlessness is no longer confined to European contexts. East Asia and North America are experiencing sharp increases in non-parenthood, prompting governments to respond with family-friendly incentives, pronatalist campaigns, or even more appeals to the role of children in national development [5–8]. The prevalence of permanent childlessness is highest among women in East Asia, i.e., Hong Kong (35%), Japan (28%), and Singapore (26%) [5]. While increasing childlessness is just one of the demographic changes in recent decades, it has emerged in public discourse - particularly in the media - as one of the most ideologically charged and widely debated issues, reflecting the broader social and political tensions that accompany changing reproductive norms.

### Media, reproductive justice, and public health

Mass media can be powerful cultural institutions that shape how societies understand a social group, demographic trends, health, and other social issues. Far from being neutral observers, media outlets may contribute to the construction of what is considered ‘normal’ and ‘healthy’, or ‘deviant’ and ‘problematic’ in public discourse. In the case of childlessness, news coverage can either challenge social stigma or reinforce discourses of deficiency, selfishness, and demographic threat. Framing analysis is a common method applied in media studies [9–12]. Framing is a central concept in health communication, as the way health information is presented in the media can shape public perceptions and influence engagement health-related behaviours [13]. The terms “frame” and “framing” are closely related, but distinct. A frame refers to the structural context that shapes human communication within specific social situations, while framing describes the process by which individuals attempt to influence one another through the way information is presented or selected as a perceived reality [10,12]. Frames highlight specific aspects of a complex issue, shaping the way audiences interpret causes, moral evaluations, and potential solutions. Frames in media communication may affect the attitudes and behaviours of their audiences [11].

Media framing analysis is a procedure used to analyse news media text, particularly applied to certain topics in health psychology, such as childlessness [14]. Media depictions of health issues often adopt either challenge or stigma frames. In a challenge frame, topics are presented with optimism, emphasizing collective resilience and the possibility of overcoming adversity. In contrast, stigma frames highlight normative exclusion, portraying affected individuals as deviant or undesirable, and encouraging avoidance rather than empathy or inclusion [15]. This can mean portraying childlessness as a private tragedy, a social epidemic, or a liberating choice. Framing also intersects with symbolic interactionism [16], which emphasizes how media participate in the ongoing negotiation of social meaning, especially for contested identities such as the non-parent.

Although often overlooked in mainstream public health models, media function as both structural and intermediary determinants of health [17]. As a cultural institution, the media influence both the conditions in which people live and the symbolic meanings attached to health-related identities, including reproductive roles. Media exposure affects health directly through its effect on psychosocial stress, stigma, and identity formation. News media serve as a major source of public understanding regarding health-related issues. Media coverage of health shapes perceptions of health risks, influence attitudes, and affect related health behaviours. As an important social determinant of health, the media play a critical role in bringing health-related issues into the public sphere. In public health discourse, media contribute to shaping how health problems are framed, including their perceived causes, responsibilities, and potential interventions. Moreover, the media play a powerful role in constructing and reinforcing particular worldviews that can become accepted cultural norms [18,19].

To deepen this perspective, this study also draws on the framework of reproductive justice [20], which expands traditional public health approaches by situating reproductive experiences within broader systems of power, inequality, and rights. Grounded in intersectional feminism and human rights, reproductive justice emphasizes not only the right to have or not to have children, but also the right to parent in safe and supportive environments. Within this framework, media are not simply sources of information but key actors in shaping whose reproductive lives are seen as legitimate, valued, or deviant. This perspective is particularly relevant in relation to childlessness, which is frequently overlooked in reproductive health discourse and policy.

By applying a reproductive justice lens, this study highlights how media representations of childlessness can either perpetuate or contest social inequalities - affecting not only social norms but also identity, individual well-being, help-seeking behaviour, and access to services for those who are involuntarily or voluntarily childless.

### Global research gaps

Despite growing scholarly interest, most studies on media representations of childlessness have focused on high income countries in the Global North. Prior analyses have been conducted in the United States (1989–2018), UK (1990–2008), Sweden (2000–2010), Norway (1999–2009), Australia (2007–2011), and Lithuania (2011–2016) [21–27]. These studies have largely applied a feminist lens, emphasizing how media reinforces or resists gender norms – particularly the framing of childlessness as a deviation from dominant ideals of femininity and motherhood. While this approach has offered important insights into stigma and autonomy, it rarely considers how such portrayals may affect health outcomes or intersect with structural determinants such as healthcare access, social support, or demographic policies.

This study adopts a public health framing, positioning media as a key social determinant of health [19]. By doing so, it shifts the analytical focus from individual identity and cultural norms to the broader implications of media narratives for population health, stigma, mental well-being, and health-seeking behaviours. This approach highlights how portrayals of childlessness may influence social inclusion, institutional response, and access to care – particularly for involuntarily childless individuals who may face barriers in health systems during reproductive or later-life stages.

There is a critical gap in understanding how news coverage of childlessness has evolved globally, particularly in non-Western or rapidly changing societies. One of the few exceptions is a recent study from Indonesia analysing two online news outlets between 2021–2023 [27]. Existing studies are, however, not only geographically limited but also typically limited in temporal scope clustering largely in the pre-COVID-19 era. Longitudinal cross-country comparisons remain rare, especially in the Global South, and large-scale, global analyses capturing the diversity of media environments and sociocultural contexts are lacking. This study addresses these gaps by offering one of the first comparative, large-scale analyses of global news coverage of childlessness, spanning a full decade (2015–2025). Notably, it captures the post-COVID-19 period, during which fertility rates, reproductive intentions, and public discourse around population issues have shifted significantly. These changes make it especially timely to investigate how media representations have evolved in the wake of pandemic-related uncertainties and resurging pronatalist rhetoric.

### Aim and contribution

This study aims to analyse how childlessness has been represented in global news media from 2015 to 2025, drawing on a public health perspective informed by discourse-sensitive thematic analysis. It explores the tone, framing, and dominant themes in media portrayals across regional contexts, with particular attention to how language contributes to the construction of stigma, social inclusion, and health-related perceptions. Our specific research questions are: (i) How is childlessness represented in news media across different countries and over the past decade? (ii) What are the dominant themes that emerge from the media portrayals of childlessness? and (iii), How is childlessness constructed in the news through the lenses of the social determinants of health framework, media framing, and discourse theory?

By conceptualizing media as a social determinant of health, the study expands conventional public health approaches to reproductive justice and well-being and situates childlessness within interdisciplinary dialogues at the intersection of media, discourse, and public health.

## Methods

### Study design

This study is a global media analysis, a qualitative research approach used to systematically examine how issues are portrayed in the media and how such portrayals reflect or shape social discourse [28]. Media analysis enables researchers to explore tone, framing, themes, and the socio-political context in which issues are communicated. In public health, media analysis has been recognized as a valuable method for understanding how media narratives influence health knowledge, attitudes, stigma, and policy debates [29,30].

While methodologically related to systematic reviews in terms of its structured search, screening, and inclusion process, media analysis applies qualitative interpretive strategies to analyse and synthesize patterns in discourse. This study uses thematic analysis informed by discourse and framing theories [9–11,31,32]. We use this approach to examine how childlessness is represented in news coverage across regions, with a focus on the public health relevance of such portrayals.

### Data sources and search strategy

News articles were identified using a multi-step search strategy that combined systematic database and manual searches. Two online databases – Google News and Factiva – were used to retrieve English-language news articles. In addition, a manual search was conducted across 12 languages (in alphabetical order): Arabic, Bahasa Indonesia, Farsi, French, German, Hindi, Korean, Chinese Mandarin, Russian, Spanish, Swahili, and Swedish. The selection of these languages was guided by the linguistic capacities of the authors and a network of collaborating researchers with native language proficiencies. The Swedish-language search was conducted with the help of the Retriever database. Together with English, these languages are spoken or understood by an estimated 5.7 billion people, representing approximately 70% of the global population. This broad linguistic coverage enhances our study’s ability to capture geographically and culturally diverse representations of childlessness. Data collection and analysis complied with the terms and conditions of the original news media sources. All sources were publicly accessible. Swedish news articles were accessed via the Retriever database through institutional access provided by Umeå University Library.

Searches were conducted for the time period between April 1, 2015 and April 1, 2025. The primary search terms used in English were ”childless” OR ”childfree”, which were then translated for use in the other languages. Factiva searches were filtered by the ”Health” section to align with our study’s public health perspective.

### Inclusion and screening criteria

**Fig 1:**
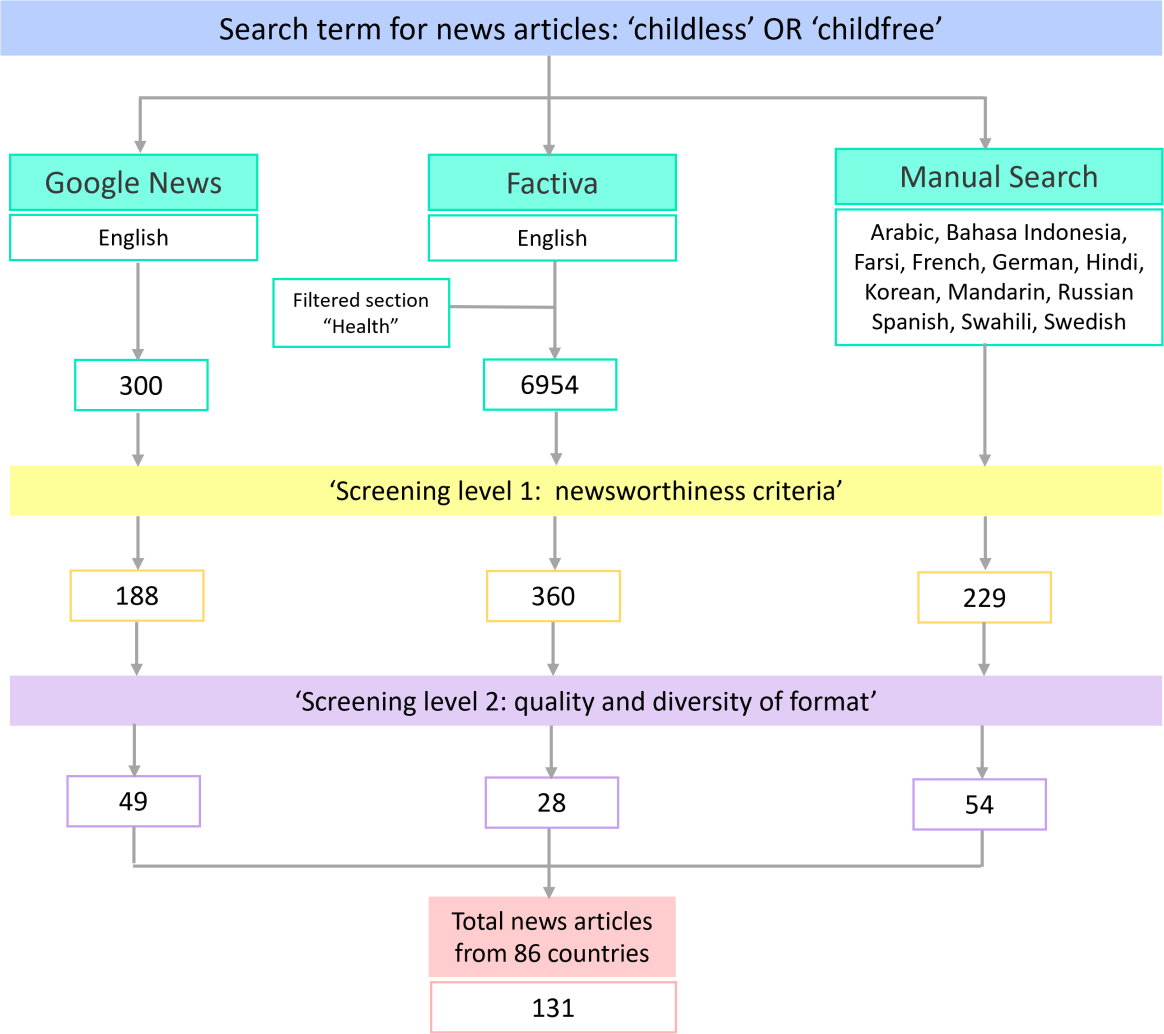
Flow diagram summarizing the search strategy and screening process.

Articles were screened in two phases, as shown in Fig 1. In the first screening, the inclusion criterion was that the news content featured elements of newsworthiness. “Newsworthiness” refers to the qualities that make a news story valuable or relevant for publication. In this study, it was defined by commonly accepted journalistic criteria such as: (i) timeliness (related to current events or recent developments), (ii) relevance (importance to the public or a specific audience), (iii) impact (potential to affect readers or societal outcomes), (iv) prominence (featuring well-known individuals or institutions), (v) human interest (emotional or relatable elements), (vi) conflict or controversy (debates, differing opinions, or societal tensions). These criteria guided the inclusion of articles that went beyond casual mentions and instead provided substance, perspective, or framing relevant to public discourse. The concept of “newsworthiness” is well-established in journalism and media studies [33].

This initial stage yielded 777 articles. The second screening evaluated article quality and diversity of format, including feature stories, breaking news, editorials, opinion pieces, investigative reports, and letters to the editor. Articles were excluded if they lacked newsworthy content related to childlessness or were duplicates. After full-text screening, a final sample of 131 articles was retained for analysis (S1 Table). Article lengths ranged from 400 to 2,500 words. Many included quotations from individuals identifying as childless, as well as commentary from policymakers, public figures, researchers, and references to empirical studies or demographic data. A total of 54 non-English articles were translated into English for inclusion in the analysis.

### Data analysis

This study employed a thematic analysis of 131 news articles on childlessness published across various global news platforms. Thematic analysis is a method for identifying, analysing, and reporting patterns of meaning across a dataset, and involves iterative coding and re-coding of text, followed by the aggregation of these codes into broader themes. We specifically used the inductive thematic analysis approach described by Braun and Clarke [34–36], which involved reflexivity and six steps of analysis: (i) familiarization with the data, (ii) coding the data, (iii) generating initial themes, (iv) reviewing and refining themes, (v) defining and naming themes, and (xi) writing up the results. Themes were reviewed and refined to ensure internal coherence and distinction from one another, and to confirm that they provided a comprehensive overview of the dataset.

Our thematic analysis was further framed with discourse sensitivity, drawing on a social constructionist perspective. It was sensitized by discourse and framing theories [9–11,32,37]. Discourse analysis examines how individuals construct their internal understanding of phenomena through discourse and has been suggested to be particularly useful when examining media texts that participate in shaping social understandings of contested topics like reproduction, family, and identity [38]*.* The combination of thematic analysis with discourse analysis has been used previously [39,40]*.* In practice, this means that our themes were re-interpreted at a deeper level with sensitivity to how language in the media performs social work - or in other words, how it shapes and reflects broader public discourses and frames around childlessness.

Discourse analysis and framing analysis may overlap, yet they remain distinct in their approaches and focus [32]. In both framing and discourse theory, analysing media also means analysing power. Framing theory emphasizes how power is used to advance particular interpretations amid a plurality of competing and negotiated meanings. In contrast, discourse theory highlights the power to hide or exclude meanings from these processes of contestation [31]. Framing theory [9–12] helps explain how certain interpretations become dominant in media narratives. We applied a discourse analytical approach to interpret how childlessness is constructed through language. In this context, childlessness is a socially constructed reality shaped by cultural conventions and media representations. Our analysis employed a critical lens, examining both the text and its broader context through comparison and multivocality. We compared the narratives and mapped the different voices within the texts, highlighting patterns of power, dominance, and inequality across multiple settings [41,42].

Coding began after an initial review of 35 articles (ca. 27% of the total sample) and initial themes emerged early in the process and were further refined and finalized until data saturation was reached after around 60 articles. However, all 131 articles were coded to ensure analytical rigour and confirm thematic recurrence. Throughout the process, reflexivity was maintained, and an audit trail of codes and themes was documented. Examples are provided in S2 Table.

### Ethical considerations

Ethical approval was not required for this study, as it relied solely on publicly accessible data from online news platforms without any interaction with human subjects or personal identifiers.

## Results

This paper uses two referencing systems: a square-bracketed Vancouver style referencing the scientific literatures listed in the References [number], and a bracketed (^*number*^) indicating the 131 news article listed in the S1 Table.

### Descriptive results

This study analysed a total of 131 news articles from 86 countries, representing all major world regions. The articles were published between 2015 and 2025 and retrieved in 13 languages, with 59% (n = 77) in English 41% (n = 54) in other languages, including Arabic, Bahasa Indonesia, Farsi, French, German, Hindi, Korean, Mandarin, Russian, Spanish, Swahili, and Swedish.

#### Geographic and temporal coverage.

**Fig 2:**
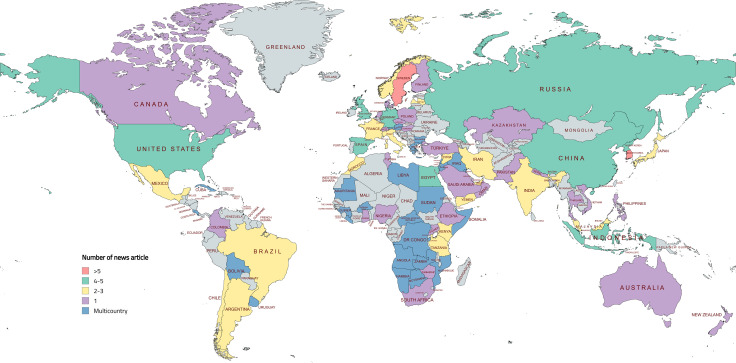
Geographic distribution of news articles on childlessness included in the analysis This figure illustrates the coverage of news articles across 86 countries. We categorized the number of news article per country into five groups: > 5, 4–5, 2–3, 1, and Multicountry. The Multicountry category includes regional coverage from Latin America and the Caribbean, Sub-Saharan Africa, Middle East and North Africa, and Europe (Created with permission from mapchart.net).

Fig 2 displays the global distribution of countries represented in this analysis. The selected articles covered every major world region, with particularly broad coverage in Sub-Saharan Africa, Europe and Central Asia, and the Middle East and North Africa (Table 1). Some articles reported on multi-country contexts, such as Arabic-speaking nations (e.g., Iraq, Jordan, Kuwait, Lebanon, Libya, Mauritania, Palestine, Somalia, and Sudan) (^*49,93*^); Latin American countries (e.g., Bolivia, Costa Rica, Cuba, and Uruguay) (^*65*^); and a group of African countries (e.g., Angola, Botswana, Burkina Faso, Burundi, Central African Republic, DR Congo, Guinea, Liberia, Mozambique, Namibia, and Zambia) (^*46*^).

**Table 1:** Geographic coverage of news articles included in the analysis, grouped by region and country (according to World Bank classifications).

No	Regions	Number of Countries	Countries
1.	Europe and Central Asia	22	Austria, Bulgaria, Czech Republic, Denmark, Finland, France, Germany, Greece, Hungary, Italy, Kazakhstan, Latvia, Netherlands, Norway, Poland, Russia, Spain, Sweden, Switzerland, Turkey, UK, Vatican City
2.	Sub-Saharan Africa	22	Angola, Botswana, Burkina Faso, Burundi, Central African Republic, DR Congo, Ethiopia, Ghana, Guinea, Kenya, Liberia, Mauritania, Mozambique, Namibia, Nigeria, Somalia, South Africa, Sudan, Tanzania, Uganda, Zambia, Zimbabwe
3.	Middle East and North Africa	16	Egypt, Iran, Iraq, Jordan, Kuwait, Lebanon, Libya, Morocco, Oman, Palestina, Qatar, Saudi Arabia, Syria, Tunisia, UAE, Yemen
4.	East Asia and Pacific	12	Australia, China, Indonesia, Japan, Malaysia, New Zealand, North Korea, Philippines, Singapore, South Korea, Thailand, Vietnam
5.	Latin America and Caribbean	9	Argentina, Bolivia, Brazil, Chile, Colombia, Costa Rica, Cuba, Mexico, Uruguay
6.	South Asia	3	India, Nepal, Pakistan
7.	North America	2	Canada, USA
**Total**		86	

#### Media sources.

The 131 articles were drawn from 101 distinct online media outlets globally. The most frequently represented outlets were the BBC (including BBC News English and BBC Arabic, 9 articles total) and Al Jazeera (6 articles). A complete list of all sources is available in S1 Table.

**Fig 3:**
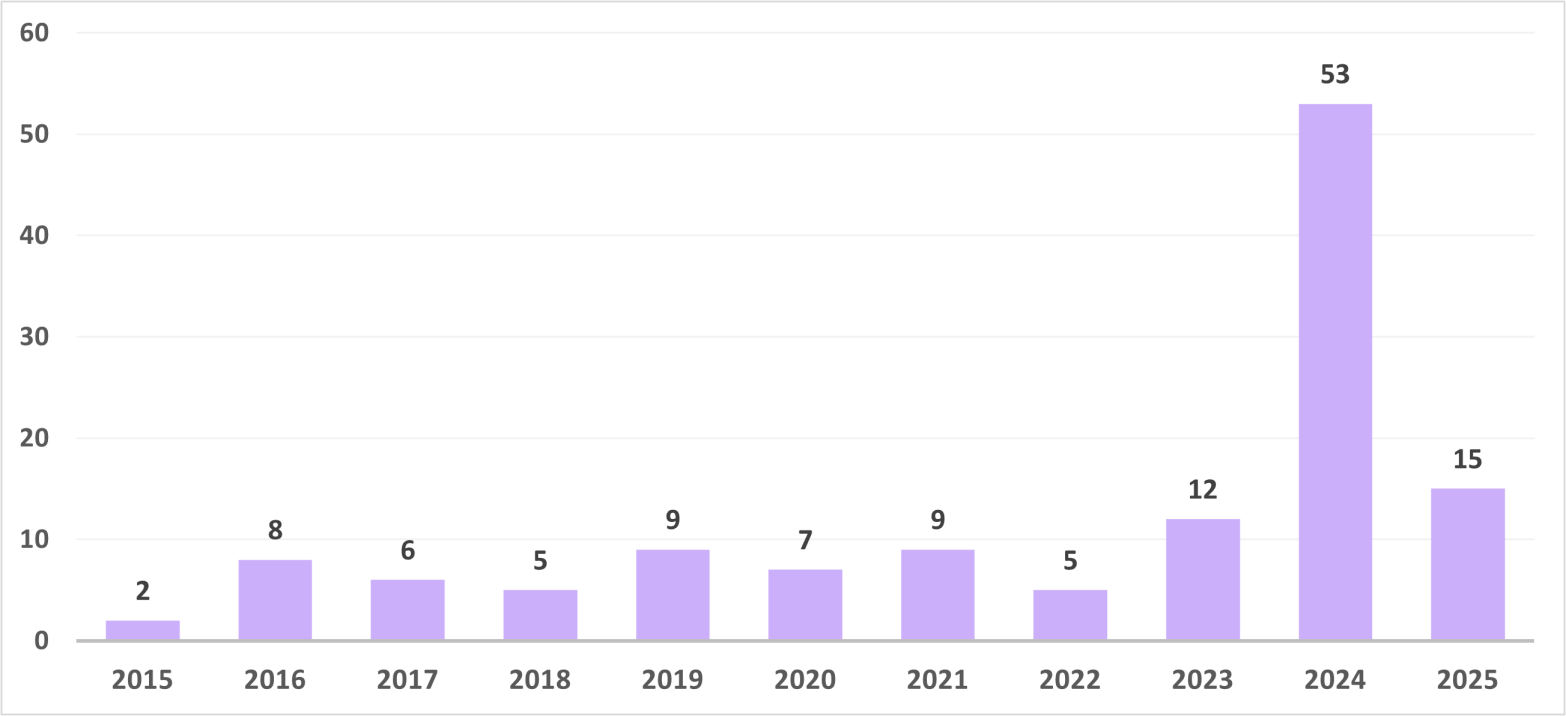
Annual distribution of news articles on childlessness published between 2015 and 2025.

Fig 3 presents the distribution of articles by publication year. The majority (77%) were published during the last five years, reflecting heightened media attention to issues of reproduction, population change, and family formation in the post-COVID-19 period. A significant number of the articles were published in 2024, likely due to heightened attention on childlessness as a campaign topic during the U.S. presidential elections and growing national concerns in Russia. These two major events may have influences news media algorithms and triggered a domino effect across various media outlets, increasing the perceived newsworthiness of the topic.

### Thematic analysis results

This study identified five major themes across 131 news articles analysed, each capturing distinct patterns in how childlessness is represented in the media. These themes reflect intersecting dimensions of reproductive autonomy, structural and sociocultural pressures, and the lived experiences of both voluntarily and involuntarily childless individual across diverse global contexts. Framed as *reproductive narratives in a changing world*, they encapsulate the tensions between individual choice, societal expectations, and state interventions in matters of childbearing. The five themes – *The guinea pig of the state; Crazy rich selfish animal lovers; No baby no cry; Bringing children into a broken world;* and *Winter regret and loneliness* – reveal how childlessness is narrated, negotiated, and contested in contemporary news discourse. Table 2 summarizes the five major themes including short descriptions of focus areas and illustrative regions where these narratives prominently appear in news coverage.

**Table 2:** Overview of the five themes identified in global news coverage on childlessness (2015–2025), summarizing focus areas and geographic emphasis.

No	Theme	Focus Areas	Illustrative Countries	References
1.	The guinea pig of the state	State control and pronatalist policies	China, Iran, North Korea, Russia, USA	(^*15, 19, 21, 23, 31, 45, 47, 51, 59, 61, 62, 67, 86, 87, 88, 89, 91, 93, 97, 98, 99, 129, 130*^)
2.	Crazy rich selfish animal lovers	Stigma and moral judgement from society and religion	Indonesia, Malaysia, Turkey, USA, Vatican City	(^*1, 3, 10, 13, 14, 18, 20, 21, 24, 33, 41, 44, 48, 49, 55, 56, 58, 65, 66, 69, 70, 72, 73, 90, 93, 94, 95, 97, 98, 99, 100, 103, 110, 113, 117, 118, 122, 123, 131*^)
3.	No baby, no cry	Empowerment and autonomy of childfree individuals	Brazil, Egypt, India, New Zealand, Philippines, Poland, Singapore, Vietnam	(^*1, 2, 9, 11, 16, 18, 20, 26, 30, 32, 35, 36, 38, 40, 42, 43, 49, 50, 53, 54, 57, 64, 68, 84, 85, 100, 111, 112, 116, 119, 120, 121, 124, 125, 126*^)
4.	Bringing children into a broken world	Environmental, economic, and psychosocial reasons for childlessness, including involuntary childlessness	Bolivia, Ghana, Kenya, Nigeria, Oman, South Korea, Syria, UAE, Uganda, Zimbabwe	(^*4, 5, 6, 7, 12, 17, 24, 25, 27, 28, 29, 34, 37, 39, 46, 60, 65, 71, 74, 75, 76, 77, 78, 79, 80, 81, 82, 96, 101, 104, 105, 106, 107, 109, 127*^)
5.	Winter regret and loneliness	Aging, loneliness, and elder care without children	Germany, Japan, Switzerland, UK, USA,	(^*8, 22, 52, 63, 83, 92, 97, 98, 102, 108, 114, 115, 122, 123, 128*^)

#### Global North vs Global South situation.

**Fig 4:**
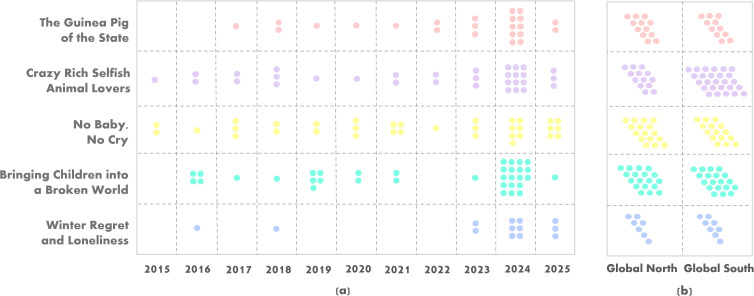
(a) Annual distribution of dominant themes in news articles on childlessness (April 2015 to April 2025), (b) Distribution of dominant themes between Global North and Global South. This distribution illustrate how certain themes may dominate during specific periods or in particular regions, reflecting how media dominance and editorial focus in different contexts can shape public discourse on childlessness.

We analysed the distribution of the themes across two regions – the Global North and the Global South. As shown in Fig 4 and Table 3, the theme *Crazy rich selfish animal lovers* appeared predominantly in news media coverage from the Global South, in contrast to its lower presence in the Global North. The remaining four themes were distributed relatively equal between the two regions. Notably, the classification of South Korea as part of the Global North influenced the distribution of the theme *Bringing children into a broken world*, as the majority of childlessness-related news coverage from South Korea (n = 8) aligned with this theme.

**Table 3:** Geographic distribution of news articles included in the analysis, grouped by Global North and Global South.

No	Regions	Number of Countries	Countries	News Article Reference
1.	Global North	29	Australia, Austria, Bulgaria, Canada, Czech Rep, Denmark, Finland, France, Germany, Greece, Hungary, Italy, Japan, Kazakhstan, Latvia, Mexico, Netherland, New Zealand, Norway, Poland, Russia, South Korea, Spain, Sweden, Switzerland, Turkey, UK, USA, Vatican City	(^*1, 3, 7, 8, 9, 11, 13, 14, 15, 16, 17, 21, 22, 23, 30, 35, 36, 37, 38, 44, 47, 48, 50, 52, 54, 60, 61, 63, 64, 74, 75, 76, 77, 78, 79, 84, 85, 86, 87, 88, 89, 90, 91, 101, 102, 103, 104, 105, 106, 107, 108, 109, 110, 111, 112, 113, 115,116, 117, 118, 119, 120, 129, 130*^)
2.	Global South	57	Angola, Argentina, Botswana, Burkina Faso, Bolivia, Brazil, Burundi, Central African Republic, Chile, China, Colombia, Costa Rica, Cuba, DR Congo, Egypt, Ethiopia, Ghana, Guinea, India, Indonesia, Iran, Iraq, Jordan, Kenya, Kuwait, Lebanon, Liberia, Libya, Malaysia, Mauritania, Morocco, Mozambique, Namibia, Nepal, Nigeria, North Korea, Oman, Pakistan, Palestina, Philippines, Qatar, Saudi Arabia, Singapore, Somalia, South Africa, Sudan, Syria, Tanzania, Thailand, Tunisia, UAE, Uganda, Uruguay, Vietnam, Yemen, Zambia, Zimbabwe	(^*2, 4, 5, 6, 10, 12, 18, 19, 20, 24, 25, 26, 27, 28, 29, 31, 32, 33, 34, 39, 40, 41, 42, 43, 45, 46, 49, 51, 53, 55, 56, 57, 58, 59, 62, 65, 66, 67,* 68, 69, *70, 71, 72, 73, 80, 81, 82, 83, 92, 93, 94, 95, 96, 97, 98, 99, 100, 114, 121, 122, 123, 124, 125, 126, 127, 128, 131*^)

Fig 4 illustrates the year-to-year distribution of dominant themes from April 2015 to April 2025. The themes *Crazy rich selfish animal lovers* and *No baby, no cry* consistenly appeared throughout the entire period. In contrast, *Bringing children into a broken world* showed the highest occurrence in 2024 compared to the other four themes. The theme *Guinea pig of the state* remained stagnant for several years before gradually increasing, particularly in the post-COVID-19 period. Similarly, the theme *Winter regret and loneliness* emerged more recently, with notable appearances in the final years of the study period.

#### The guinea pig of the state.

This theme highlights how population policies intersect with reproductive health and rights in news media from several countries. Declining fertility rates have become a pressing demographic concern not only in high-income countries but also increasingly in middle- and low-income contexts. As portrayed in news coverage, political leaders—from China and Russia to the United States and North Korea—have urged women to have more children to sustain national populations, often linking motherhood to patriotic duty and traditional gender roles:

*“When all mothers clearly understand that it is patriotism to give birth to many children and do so positively, our cause of building a powerful socialist country can be hastened faster.”* (Kim Jong-un, North Korea) (^*59*^)

While concerns over economic growth and social security may justify policy attention to fertility rates, these pressures are overwhelmingly directed at young women, reinforcing gendered expectations around reproduction. In Russia, for example, the government has attributed the country’s population crisis to the spread of ‘childfree ideology’, sidestepping more political sensitive drivers such as war and economic security. A notable example of pro-natalist messaging is Russian reality television show that rebranded from *“Pregnant at 16”* to *“Mom at 16”*, portraying teenage motherhood as a patriotic contribution to the nation (^*88*^). This discursive change illustrates how reproductive autonomy is increasingly undermined by state narratives, especially as access to contraception and abortion becomes more restricted under Kremlin policies:

“*A woman is like an incubator that delivers new warriors, new people to be exploited, new people for the government. We see that a lot in propaganda and on billboards about how you’re going to give birth to a soldier who is going to protect our land*.” (Washington Post, Russia) (^*89*^)

Governments in various countries, including China, France, Germany, Norway and Sweden have introduced financial incentives such as subsidies and tax benefits to support childbearing and reduce the burdens of parenthood (^*13,19,38,50,119*^). While these family-friendly policies aim to improve work-life balance, they have not been sufficient to reverse declining birth rates or address the deeper structural or gendered barriers that discourage childbearing.

Reproduction is increasingly framed not only as a private or family matter but as a matter of national importance. In 2021, China formally replaced its two-child policy with a three-child policy, a dramatic shift that generated mixed reactions among women (^*45*^):

*Liu, meanwhile, said the women she has spoken to have described a feeling of whiplash at the shifting government family policies they’ve seen in their lifetimes. The first generation born under the one-child policy, they’re now also the first people in a generation being asked to have second children. “Single girls were the only daughters, and they feel like they are trapped in an experiment,” Liu said.* (Caixing Global, China) (^*19*^)

Such abrupt policy changes, especially when communicated through nationalist rhetoric, may carry mental health implications for women who feel caught between personal aspirations and the perceived duty to reproduce. In Iran, for instance, news report that the government has restricted access to contraception and vasectomies at public hospitals as part of its efforts to raise the national population:

*Iran has limited the provision of family planning services at state-run hospitals as it tries to boost its population size. Vasectomies will no longer be carried out at state-run medical centres and contraceptives will only be offered to women whose health might be at risk. Supreme Leader Ayatollah Ali Khamenei has been calling for people to have more children, saying he wants the current population of 80 million to grow to 150 million.* (BBC, Iran) (^*31*^)

Although the degree of pro-natalist policy varies by country, a shared objective persistently mentioned in the news: to meet the replacement rate threshold of 2.1 children per woman. This discursive approach frequently places the burden of demographic sustainability on individual women, positioning them as instruments of state-building rather than autonomous decision-makers.

#### Crazy rich selfish animal lovers.

This theme shows how media representations of childless individuals reflect societal, religious, and cultural discourses often depict voluntary childlessness – especially among women – as selfish, immoral, or unnatural. Across different country contexts, choosing not to have children is depicted not as a valid life choice, but portrayed as a deviation from social norms tied to femininity and masculinity, morality, and religious duty:

*“Today … we see a form of selfishness. We see that some people do not want to have a child. They have dogs and cats that take the place of children. This may make people laugh but it is a reality. A denial of fatherhood and motherhood diminishes us, takes away our humanity.”* (Pope Francis, Vatican City) (^*48*^)

Several news articles portray that Pope Francis has repeatedly linked childlessness to a loss of humanity, reinforcing the moral authority of parenthood. During a 2024 visit to Indonesia, he praised families with more than three children as role models, despite the country’s Total Fertility Rate (TFR) already being close to replacement level at 2.18 (^66^). Similarly, in Malaysia, religious leaders have declared that viewing children as a burden contradicts Islamic teachings and traditional family values (^*69,70*^).

In other settings, childlessness has become a flashpoint in political discourse. In the United States, reproductive choices and motherhood were used as rhetorical device during the 2024 election. Republican Vice-Presidential candidate, JD Vance, infamously referred to Kamala Harris as a “childless cat lady”, provoking widespread backlash for his derogatory characterization of women without biological children:

*The former Trump White House press secretary’s argument that children serve as reminders of what matters in an election, which alluded to the vice president’s lack of biological kids, was reminiscent of repeated comments made by Trump’s running mate, Sen. JD Vance. The Ohio Republican has received criticism for his past remarks that the US was being run by “childless cat ladies who are miserable at their own lives and the choices that they’ve made.”* (CNN, USA) (^*90*^)

Social norms often equate happiness and success with parenthood. Those who define fulfilment differently—pursuing career, freedom, or non-parenting lifestyles—are seen as non-conforming or incomplete. Even highly accomplished women, such as Kamala Harris, are frequently portrayed as lacking something essential. This sentiment is echoed not only by the public (^*7,10*^), but also by political leaders around the world:

*“Rejecting motherhood means giving up on humanity. I would recommend having at least three children. The fact that a woman is attached to her professional life should not prevent her from being a mother. A woman who rejects motherhood, who refrains from being around the house, however successful her working life is, is deficient, is incomplete.”* (Erdogan, Turkey) (^*3*^)

Couples who voluntarily remain childless are often labelled as DINKs (Double Income, No Kids), with assumptions of selfishness or excessive wealth. Such narratives were reflected in news from Indonesia, where societal and family expectations framed childless couples as morally suspect and financially obligated to support others (^*41,59*^):

*“Every time we are at my mother-in-law’s house, they will ask us for money. My mother-in-law encourages this because she thinks we have a lot of money. Annoying, right?”* (The Jakarta Post, Indonesia) (^*41*^)

Across many societies, childless individuals continue to face social exclusion and are rarely recognized as legitimate members of the normative life course (^*9,18,24,41,42,56,73*^). In summary, this theme illustrates how religious and cultural discourses – reinforced by the political narratives explored in Theme 1 – converge to stigmatize childlessness and constrain prevailing definitions of fulfilment, morality, and even citizenship.

#### No baby, no cry.

Building on the first theme that highlighted state control and societal stigmatization of childlessness, this theme captures media representations of childless individuals exercising agency and reproductive autonomy. It portrays how individuals, especially childfree women, actively redefine fulfillment and happiness outside of parenthood, resisting dominant pro-natalist narratives and affirming childlessness as a self-determined life choice:

*“We don’t want to give up our current life to raise children. We’ve seen couples around us do it for the sake of it, and it doesn’t look like fun in most cases.”* (The Swaddle, India) (^*18*^)

Despite facing social marginalization, childless individuals in diverse contexts—including the Philippines, Vietnam, and New Zealand—expressed a sense of liberation and empowerment through their decision (^*9,32,57*^). For many, choosing not to have children was associated with greater psychological well-being and the freedom to pursue meaningful goals outside of traditional family roles:

*“As a woman who chooses to be childless, I’m viewed as a woman who isn’t fulfilling her potential, but I am fulfilling my potential as a human being. I feel complete and I am not any less of a woman without a child.”* (Egyptian Streets, Egypt) (^*42*^)

These representations challenge normative constructs of marriage and reproduction, particularly in cultures where procreation is seen as central to personal and social fulfillment. The motivation to marry, for instance, was depicted less as an obligation to reproduce and more as a path to mutual growth and personal choice:

“*Now, many young adults argue they are marrying for intrinsic reasons. The purpose of getting married is for self-fulfillment rather than extending your family or meeting your extended family’s expectations. Unless there’s an intrinsic love for children, the couple may think children make it so difficult to balance family commitments and work commitments, they may decide not to have children.”* (The Straits Times, Singapore) (^*43*^)

Some individuals emphasized the importance of bodily autonomy by opting for sterilization, particularly in settings where reproductive rights are politicized or gender norms are rigid. Such examples were reported among women and men in Singapore, Poland, and Brazil (^*43,54,125*^).

*“THE PROCEDURE TOOK 15 MINUTES, AND NOT EVEN A SCAR WAS LEFT BEHIND. He described his experience in a Facebook post, which quickly went viral and reached tens of thousands of people. In addition to congratulations or expressions of admiration for his courage and responsibility, there was also criticism. It read that he was the antichrist, a hedonist, who wants to “shag without consequences,” and that he would yet regret his decision.* (Newsweek, Poland) (^*54*^)

In contrast to the earlier themes where reproductive behaviour is shaped or constrained by external expectations this theme surfaces counter-narratives that reassert the right to remain childfree as a legitimate lifestyle. This theme highlights how voluntary childlessness can be a deliberate health-promoting, and identity-affirming choice, even in the face of societal resistance. While these individuals reject parenthood as a path to fulfillment (or build up alternative identities for fulfillment), the next theme captures how individuals are portrayed with anxieties, such as climate change, conflict, and economic precarity, shaping the decision not to have children.

#### Bringing children into a broken world.

Building on the previous theme’s emphasis on personal agency in reproductive decisions, this theme explores how broader structural and existential anxieties – such as climate change, war, economic instability, and gender inequality – influence decisions to remain childless. It highlights media portrayals of individuals navigating the emotional and ethical complexities of parenting in what many perceive as an increasingly unlivable world.

One example of the depicted anxieties is the rise of the self-identified GINKs (Green Inclined, No Kids) in France – individuals who reject parenthood on ecological grounds, citing concerns about overpopulation and environmental degradation (^*35*^). Climate anxiety, however, is not confined to high-income countries; concerns about resource scarcity and planetary instability are echoed globally, including in fragile and conflict-affected settings.

*“It is a great crime to marry and have children under wartime conditions, to expose them to fear and death, to feed them with people’s charity, and to heal them with our tears and sorrows. It is a crime to make them suffer from the heat of summer and the cold of winter, from harsh conditions, from deprivation of education, and from misery… These are the most basic rights of children and human rights, so why do we participate in their misery?”* (BBC Arabic, Syria) (^*39*^)

Psychological distress emerged as a powerful deterrent to childbearing. Women in war zones and politically unstable contexts cited fear, trauma, and future uncertainty as reasons to delay or reject parenthood. Yet similar sentiments were expressed in high-income contexts as well. For example, women in South Korea – home to the world’s ultra-low fertility rate (TFR 0.68 in 2024) – linked their choice not to have children to intense social pressures, economic precarity, and lack of psychological well-being (^*79*^):

*“I’ve had to compete endlessly, not to achieve my dreams, but just to live a mediocre life. It’s been so draining. Korea is not a place where children can live happily.”* (BBC, South Korea) (^*74*^)

This “shadow of a life without leisure”, as it was described in Korean media (^*76*^), reflects a deep gendered asymmetry. As depicted, women bear disproportionate costs of reproduction—financial, emotional, and professional—which are compounded by unequal social expectations. News discourses from Bolivia and other countries similarly reflect how gender inequality in both the labour market and the home diminishes the appeal of parenthood:

*“The transition to motherhood continues to be very costly because it reveals strong gender inequalities and today women are less willing to start a family in conditions that seem asymmetrical to them.”* (El Tiempo, Bolivia) (^*65*^)

Gender inequality in both the labour market and domestic sphere can diminish the perceived benefits of marriage and motherhood for young women. Choosing childlessness, therefore, may serve as a form of self-preservation or resistance in inequitable systems that demand disproportionate self-sacrifice.

Beyond voluntary decisions, the theme also includes news representations of involuntary childlessness. Several articles, particularly from African and Middle Eastern media (^*4,5,6,12,24,25,34,96*^), covered infertility and its sociocultural consequences – ranging from stigma and ostracism to physical violence:

*“In some cultures, childless women still suffer discrimination, stigma and ostracism. An inability to have a child or to become pregnant can result in being greatly isolated, disinherited or assaulted. This may result in divorce or physical and psychological violence.”* (All Africa, Nigeria) (^*5*^)

Moreover, “social infertility” emerged as a concern – cases where individuals may not face medical infertility but become childless due to delayed childbearing, lack of supportive partnerships, or economic and political constraints (^*72,101*^).

In sum, this theme illustrates how deeply interconnected reproductive decisions are with local and global crises, structural inequities, and individual well-being. While some choose to remain childless for ideological or practical reasons, others face involuntary barriers rooted in injustice and instability. The final theme turns towards the long-term implications of such decisions, particularly the experiences of aging without children.

#### Winter regret and loneliness.

The last theme captures how news media depict the evolving life-course experience of childlessness, and changes that occur in older age. While earlier themes highlighted reproductive choices and societal pressures, this theme centers on the social and emotional vulnerabilities that may emerge in later life among those aging without children, including feelings of regret, loneliness, and uncertainty about care and support in old age.

News potrayed the later stage of life—like the winter as the last season of the year—as bringing a growing sense of regret and loneliness for some older adults without children. Aging without children is depicted as an emerging public health concern, linked to limited access to care and increased mental health risks. Several news articles from Germany, the UK, and Switzerland shared the perspectives of older men reflecting on their childless lives (^*52,102,108*^).

*“As a childless man of 64 who has always wanted children, here’s the truth: it’s an isolating and at times crushing experience. There’s a unique grief that comes with unintentional childlessness, but men like me are assumed to be happy, and constantly told that we’re “free”. You wouldn’t tell a woman the same. People look at you like you’re strange when you reveal that you’ve always dreamed of being a dad.” (The Telegraph, UK)* (^*102*^)

Despite rising life expectancy and increasing numbers of older adults, many countries lack sufficient elder care infrastructure. The so-called “elderquake” (^*27*^) has revealed a demographic shift that is not matched by adequate policy planning. In most societies, care responsibilities are still largely placed on the family, leaving childless older adults especially vulnerable:

*“Carsten Lorentz is also at a point where he is beginning to look back on his life. He will retire in two years’ time. The fact that he won’t be passing on his genes doesn’t bother him. Above all, he is afraid of the emptiness.”* (Süddeutsche Zeitung, Germany) (^*108*^)

A news article from the United States drew attention to “elder orphans”—seniors without close relatives or family caregivers to help with health or legal decisions. These individuals often struggle to access appropriate support systems and remain unheard in policy debates (^*8*^). In the UK, the number of childless older adults living with dementia is expected to rise, further underscoring the need for responsive care models (^*22*^).

*“As you get older, you tend to become more isolated. This type of community aims to bring more social interaction and not allow the elderly to be left out. We see that the population is getting older, and there is this fear. Community life can be beneficial for health and even for gaining a few years of life.”* (CE NoticiasFinancieras, Brazil) (^*92*^)

Positive examples of community-based responses came from Brazil and China, where older adults form support networks that reduce isolation and promote mutual care (^*83,92*^). In Spain, social expectations still emphasize family caregiving for older relatives (^*14*^).

*“Women tend to live longer than men, and that explains why many elderly women live alone, but in recent years, a growing number of men have no family members or relatives to rely on.”* (The Asahi Shimbun, Japan) (^*115*^)

Japan, with the highest life expectancy in the world, is experiencing a growing number of childless elderly men who live alone and without social support. This so-called *“elderquake”*—a demographic shift driven by aging and declining fertility—is prompting national concern over isolation and the urgent need for improved care provision for older men living alone (^*115*^).

In summary, this theme presents the often-silenced vulnerabilities of childless aging. It highlights how social structures built around family-based caregiving fail to meet the needs of a growing population aging without children, raising urgent questions about health, social inclusion, and policy preparedness.

### Thematic discourse analysis results

**Fig 5:**
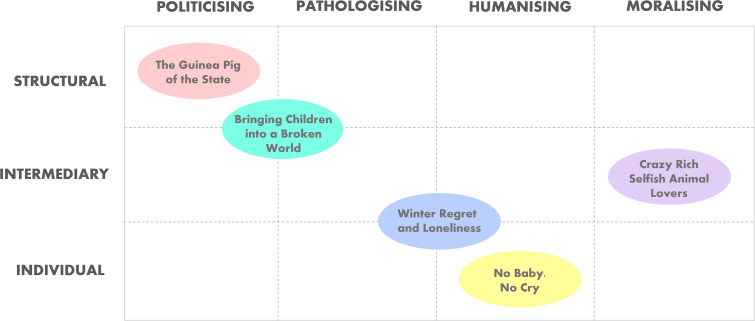
Situational map of global media narratives on childlessness, based on 131 news articles from 86 countries (2015–2025). The map visually organizes five dominant themes according to their primary narrative function (politicising, pathologising, moralising, humanising) and the social level at which they operate (structural, intermediary, individual). Themes often span multiple narrative functions, reflecting overlapping discursive strategies that shape public understandings of childlessness. The mapping was developed through a comprehensive review of all narratives and emerging themes from the total sample, guided by Social Determinants of Health framework, as well as framing and discourse theory.

Across 131 news articles, five interrelated themes reveal how childlessness is portrayed, framed, and debated in global media discourse. These media representations illustrate the complex interplay between state narratives, sociocultural expectations, and individual agency in shaping public understanding of reproductive choices. Our discourse-aware re-interpretation of the five themes identified four narrative functions – politicising, pathologising, moralising, and humanising - that represent discursive strategies through which media texts construct social realities, identities, and power relations. Fig 5 visually maps the five themes according to their primary narrative function and the social level at which they operate, i.e., structural, intermediary, or individual, based on the Social Determinants of Health model. These thematic representations indicate different discourses: an institutional discourse and state-centric framing that constructs childlessness not as a personal issue but as a collective, governable concern, drawing attention to political ideologies (e.g., pronatalism, nationalism); a medicalised discourse that contributes to biopolitical control—shaping public attitudes by embedding childlessness within discourses of deficit, illness, or risk; a normative social discourse about the “natural” life course, femininity and masculinity, reproduction as responsibility or moral failure; and a personalised, experiential discourse that invites identification and reframing of stigma, often creating discursive space for resistance or alternative understandings.

From pro-natalist policies that politicize women’s bodies (*The guinea pig of the state*), to moralising cultural and religious discourses that depict childlessness as selfish (*Crazy rich selfish animal lovers*), to narratives that humanise individual autonomy and fulfilment outside of parenthood (*No baby, no cry*), the media both mirrors and reinforces dominant norms. Coverage also highlights a pathologising lens, showing how concerns about environmental collapse, economic precarity, and social instability influence reproductive choices (*Bringing children into a broken world*), and how aging without children is increasingly framed as a public health concern (*Winter regret and loneliness*). Taken together, these themes reflect how reproductive narratives are constructed and contested in the media within a rapidly changing world.

## Discussion

### Framing childlessness in the media: Implications for public health and equity

The five themes reflect both the reasons for childlessness and the lived experiences of childless individuals, capturing the core meanings conveyed in the news narratives. The themes identified in global news portrayals across 86 countries, illustrate how the media operates as a social determinant of health. These portrayals emerge across structural, intermediary, and individual levels, shaping and reinforcing reproductive narratives through a public health lens. The media is not a neutral mirror of society–it is an active agent in constructing social reality and potentially shaping health outcomes. It reflects and reinforces dominant societal values, shapes public attitudes and health behaviours, and affects access to care and support. As a central actor in public discourse, the media determines which issues gain visibility and how they are framed—often producing meanings that extend beyond objective reality [19,43].

This study found that media coverage of childlessness has expanded both geographically and over time, accompanied by a wide range of tones and narrative framings. Childlessness was variously constructed as a threat to national interests, reflecting a *politicising* function that frames reproductive decisions in terms of state needs and demographic anxieties; as a deviation from moral or cultural norms, demonstrating a *moralising* function that upholds traditional ideals of family and parenthood; as a source of risk or dysfunction, pointing to a *pathologising* function that links childlessness to personal or societal deficiency; and as a legitimate, self-defined life path, embodying a *humanising* function that affirms autonomy and diverse life choices. These narrative functions are central to how the media shapes public understanding of reproductive behaviour and the social status of those who are childless. These framings contribute to a constructed reality in which childlessness is made more visible—whether framed as a social challenge or individual stigma [15,44].

Comparable patterns have been observed in media representations of other public health issues, such as HIV/AIDS, mental illness, and obesity [44–46], where negative media representations have possibly contributed to stigma, discrimination, and exclusion from health services. In this context, the media can function either to reinforce social exclusion and inequity or to promote inclusion and recognition, shaping both health behaviour and public perception. This dual role highlights the media’s capacity to either exacerbate or reduce health disparities, depending on how it constructs reproductive narratives and whose voices it amplifies.

### Mapping news representations of childlessness across levels and narrative functions

Building on the framework of social determinants of health [43], the themes in this study reflect how media narratives operated across structural, intermediary, and individual levels. These framings do not merely communicate information; they perform social and political work—assigning moral value, legitimising state policies, or affirming individual autonomy.

The four dominant media functions identified–politicising, moralising, pathologising, and humanising—serve to direct not only what the public should think about childlessness, but also how to interpret its meaning and legitimacy. As Kim & Willis (2007) argue [45], framing shapes perception by establishing what counts as a problem, whose voices are prioritised, and which solutions are considered acceptable.

By mapping each theme across levels of influence and narrative function (Fig 5), this study highlights how media actively constructs reproductive health as a terrain of political and moral contestation. This layered framing helps explain why childlessness becomes a socially visible–and often polarized–issue, with consequences for both individual well-being and policy discourse.

### Reframing global media narratives of childlessness: Intersections and implications

As shown in Fig 5, media narratives were analysed along two intersecting axes: levels of influence (structural, intermediary, individual) and narrative functions (politicising, moralising, pathologising, humanising). While some themes aligned clearly with specific levels and functions, others were more complex, cutting across multiple domains—reflecting deeper entanglements between structural systems, social norms, and individual experiences.

Some themes reflected distinct roles of the media in shaping reproductive narratives.

For example, the theme *The guinea pig of the state* exemplifies a structural-level, politicising function. In this narrative, national media—particularly in countries such as China, North Korea, Iran, and Russia—framed declining birth rates as existential threats, positioning women’s reproductive choices as matters of state interest. These portrayals closely parallel how other public health issues, such as the COVID-19 pandemic and lockdown [47,48], or vaccination and abortion, have been politicized to serve nationalist agendas, as shown in prior studies [49,50]. When reproductive autonomy is co-opted by demographic discourse, it can reduce trust in health governance and contribute to societal pressure, misinformation, and anxiety. Such resurging pronatalist rhetoric - visible across diverse political systems - reflects a broader global trend in which fertility becomes a symbol of national strength and demographic anxiety. Our findings highlight how these media narratives not only reproduce gendered expectations but also normalize state intervention in intimate reproductive decisions.

The theme *Crazy rich selfish animal lovers* illustrated the intermediary level and moralising function. Here, media narratives—particularly in Malaysia, Vatican City, and Turkey—cast childfree individuals as selfish or deviant, often drawing upon religious or cultural scripts. Women were disproportionately targeted, represented as incomplete without children and criticized for valuing careers or pets over parenthood. Such portrayals resonate with earlier findings that religious norms and benevolent sexism reinforce parenthood as a moral obligation, with non-conformity provoking social sanction [51,52]. Likewise, the media’s role in moralising reproductive health issues, such as transnational commercial surrogacy, has revealed prevalent stereotyping, indicating that media selectively influences its readership [53]. The media thus plays an active role in moralising reproductive behaviour and reinforcing gendered expectations. This theme, which appears more prominently in the Global South than Global North, may reflect underlying structural inequalities – particularly in health, economic, and environmental domains. The polarization of North and South is often rooted in disparities in economic capacity, with health inequalities closely intertwined with economic inequalities [54].

In contrast, *No baby, no cry* represented the individual level and a humanising function. This theme featured stories that affirm voluntary childlessness as a valid and fulfilling choice. Media outlets in Egypt, Sweden, and New Zealand portrayed childfree individuals as autonomous, reflective, and content—often emphasizing self-defined well-being and identity. Previous studies that amplify the perspectives of childless individuals on the overwhelming cultural pressure of parenthood suggest that motherhood can be viewed both as a “myth”—where children are presumed to provide fulfilment—and as a “mystique”—where women are believed to be biologically “wired” to want children. These depictions echo studies showing that non-parents often report similar or higher levels of life satisfaction than parents [8,55]. Through personalisation and empathy, the media can humanise marginalised reproductive choices and challenge dominant norms.

### Cross-level and multi-functional themes: Complexity and context

Two themes proved more complex, spanning multiple levels and narrative functions. *Bringing children into a broken world* combined structural and intermediary concerns—such as climate anxiety, economic insecurity, and gender inequality—with individual mental health considerations. In fragile states like Syria and in economically advanced but socially pressured settings like South Korea, decisions not to have children were portrayed as both rational responses to crisis and sources of internal conflict. In African contexts, the media framed infertility as a health burden while invoking political and public figures to call for improved services—thereby merging pathologising and politicising functions. This complexity is also observed in other health narratives, such as surrogacy or parenting in migration contexts, where emotional, economic, and policy dimensions intertwine [53,56]. Whereas this theme employed the ethic of empathy to humanise individual experiences, journalism also relies on the ethic of solidarity to highlight shared conditions of social injustice across different contexts [57]. Media use politicisation techniques to expose community-wide constraints that prevent individuals from overcoming marginalisation, while representing diverse perspectives on structural and intermediary-level solutions to social injustice.

The theme *Winter Regret and Loneliness* similarly straddled the intermediary and individual levels and reflected both pathologising and humanising tones. Media in Japan, Germany, and the UK highlighted isolation and unmet care needs among older childless adults, often portraying them as at risk or socially invisible. At the same time, some articles emphasized resilience and community-based alternatives. This dual framing aligns with gerontological studies showing that childless older people are frequently left out of policy agendas and public discourse [58,59]. That this theme spans both intermediary and individual levels suggests that aging without children is both a personal challenge and a societal failure in care infrastructures. The complexity of this theme may reflect the intensifying global challenge of population aging and the unpreparedness of many health and welfare systems to support diverse aging trajectories.

Together, these media framings of childlessness reveal the multi-layered nature of discourse surrounding childlessness and demonstrate how the media functions not only as a reflector of dominant values but as a powerful cultural institution in shaping public understanding and health-related experiences. The social determinants of health framework, which emphasizes the influence of social and economic conditions on health outcomes, traditionally addresses the biosphere and social sphere but omits the infosphere. In the contemporary digital era, however, the distinction between online and offline environments has become increasingly blurred, with individuals effectively living “onlife”—a hybrid existence shaped by both physical and digital realities [43,60]. Recognizing the information environment and communication infrastructure as integral components of the social determinants of health framework is essential for mitigating health disparities in the digital age. For example, during the COVID-19 pandemic, misinformation disproportionately impacted ethnic minority communities. As the WHO noted, populations worldwide were contending not only with a pandemic but also with an “infodemic”—an overabundance of information, including false or misleading content, that complicated public health responses [60–62].

### Strengths and limitations

To our knowledge, this is the first study to examine media representations of childlessness in the global context, drawing on a sample of 131 news articles from 86 countries over a ten-year period (2015–2025). The data collection employed a multi-method approach using three distinct strategies and covered 13 languages, offering a broad geographic and linguistic representation. The lead researcher’s professional background in journalism added value during the screening process, particularly in assessing the newsworthiness and quality of articles selected for analysis.

However, this study contains several limitations. First, the screening and selection of articles were conducted by a single researcher, which may have introduced bias or led to the omission of relevant sources. Particularly, the identification and labelling of themes may have been shaped by the researchers’ positionality, potentially influencing how narratives were framed – despite the application of reflexivity and systematic coding procedures. To strengthen our manuscript’s validity – both linguistically and analytically – all co-authors contributed to a thematic verification process. Each was asked to select three articles in the language or country they had previously supported and assess as native speakers whether one or more of the identified themes were reflected in the content. This step served as an additional check to ensure trustworthiness of theme identification across linguistic and regional contexts.

Although this study did not adopt a full discourse analytical approach, the identification of four narrative functions – politicising, pathologising, moralising, and humanising – reflects a discourse-sensitive interpretation. These functions illustrate how media narratives do more than describe childlessness; they actively shape its meaning through moral, political, medical, and empathetic framings. As such, this study does not just identify themes, but interprets what they do in public discourse.

Third, the translation of non-English articles relied primarily on Google Translate. While this allowed to broad linguistic coverage, it may have resulted in the loss of nuance, tone, or culturally specific references that would ideally be captured through native-speaker validation. In addition, non-dominant languages and non-digitized perspectives, especially from low-income or rural areas, may be underrepresented.

Lastly, the findings should be interpreted with caution and not generalized to specific national contexts. Media portrayals of childlessness are shaped by varying sociopolitical, cultural, and religious environments, which influence whether representations are positive, negative, or ambivalent. Moreover, media gatekeeping and editorial policies, i.e., decisions about what gets published – are shaped by institutional agendas and journalistic norms. These processes can reinforce dominant narratives while marginalising others, including LGBTQ+ individuals, people with disabilities, migrants, or rural communities. Although our analysis identifies these gaps, the available news coverage does not allow us to fully examine how intersecting identities (e.g., sexuality, disability, gender, or migration status) shape media constructions of childlessness. As such, the data in this work may reflect dominant discourses more than the full diversity of lived realities.

## Conclusions

Childlessness is not merely a personal preference but a complex, multi-layered phenomenon shaped by sociocultural expectations, gender norms, and individual agency. This study confirms that global news media representations of childlessness intersect with key public health concerns, employing varied narrative functions to shape public discourse. The media’s role as a social determinant of health is crucial – not only for disseminating health information but also for influencing social norms, shaping health behaviours, and informing public policies.

However, disparities in access to accurate, credible, and inclusive media content across socioeconomic groups have the potential to widen the information gap, thereby contributing to broader well-being gaps. Within the health sector, these disparities may further exacerbate existing inequalities healthcare access, utilization, and outcomes – particularly for those of lower socioeconomic status. By reinforcing dominant ideologies while marginalizing diverse experiences, media representations risk perpetuating structural imbalances and social exclusion. Conversely, more inclusive and diverse media framing may offer a pathway to greater equity, recognition, and social inclusion for individuals who are voluntarily or involuntarily childless.

These findings demonstrate that media does not merely reflect reproductive realities but actively constructs them. As a social determinant of health, the media shapes public understanding, influences health behaviours, and sets the tone for policy debates. Recognizing this, there is a need for stronger media literacy initiatives to help audiences critically engage with dominant narratives, and for public health communicators to work more proactively with media outlets to promote inclusive, stigma-free representations of childlessness. Together, these efforts can support more equitable health communication and contribute to broader goals of reproductive justice and social inclusion.

## Supporting information

S1 Table List of news articles included in the analysis. (DOCX)

S2 Table Illustrative audit trail documenting the thematic analysis process. (DOCX)

## References

[1] Gouni O, Jarašiūnaitė-Fedosejeva G, Kömürcü Akik B, Holopainen A, Calleja-Agius J. Childlessness: concept analysis. Int J Environ Res Public Health. 2022;19(3):1464. doi: 10.3390/ijerph19031464 35162484 PMC8834711

[2] Kreyenfeld M, Konietzka D. Childlessness in Europe: contexts, causes, and consequences. Springer Nature; 2017.

[3] World Health Organization. 1 in 6 people globally affected by infertility. WHO; 2023 [cited 2025 May 22]. Available from: https://www.who.int/news/item/04-04-2023-1-in-6-people-globally-affected-by-infertility

[4] Miettinen A, Rotkirch A, Szalma I, Donno A, Tanturri ML. Increasing childlessness in Europe: time trends and country differences. Families and Societies. Working paper series. 2015.

[5] Sobotka T. World’s highest childlessness levels in East Asia. Pop Soc. 2021;595(11):1–4.

[6] Jiang Q, Zhang C, Zhuang Y, Jiang Y, Zhang X. Rising trend of childlessness in China: analysis of social and regional disparities with 2010 and 2020 census data. BMJ Open. 2023;13(5):e070553. doi: 10.1136/bmjopen-2022-070553 37236662 PMC10231016

[7] Choi KH, Qian Y. The rise of the childless single in South Korea. J Fam Theory Rev. 2023;15(3):526–41. doi: 10.1111/jftr.12507

[8] Hummer H. Motherhood myths and mystiques: how childless women navigate cultural beliefs about motherhood. J Marriage Fam. 2024;86(4):1098–118. doi: 10.1111/jomf.12996

[9] Entman RM. Framing US coverage of international. J Commun. 1991;41(4):52.

[10] Entman RM. Framing: towards clarification of a fractured paradigm. In: McQuail’s reader in mass communication theory; 1993. p. 390–7.

[11] Chong D, Druckman JN. Framing theory. Annu Rev Polit Sci. 2007;10(1):103–26.

[12] D’angelo P. Framing theory and journalism. In: The international encyclopedia of journalism studies. Wiley; 2019. p. 1–10. doi: 10.1002/9781118841570.iejs0021

[13] Guenther L, Gaertner M, Zeitz J. Framing as a concept for health communication: a systematic review. Health Commun. 2021;36(7):891–9. 31996044 10.1080/10410236.2020.1723048

[14] Giles D, Shaw RL. The psychology of news influence and the development of media framing analysis. Soc Pers Psychol. 2009;3(4):375–93. doi: 10.1111/j.1751-9004.2009.00180.x

[15] Smith R. Media depictions of health topics: challenge and stigma formats. J Health Commun. 2007;12(3):233–49. doi: 10.1080/10810730701266273 17497378

[16] Stryker S. From Mead to a structural symbolic interactionism and beyond. Annu Rev Sociol. 2008;34(1):15–31.

[17] WHO Commission on Social Determinants of Health, World Health Organization. Closing the gap in a generation: health equity through action on the social determinants of health: Commission on Social Determinants of Health final report. World Health Organization; 2008.

[18] Dhanani LY, Franz B. The role of news consumption and trust in public health leadership in shaping COVID-19 knowledge and prejudice. Front Psychol. 2020;11:560828. doi: 10.3389/fpsyg.2020.560828 33192827 PMC7642623

[19] Henderson L, Hilton S. The media and public health: where next for critical analysis? Crit Public Health. 2018;28(4):373–6. doi: 10.1080/09581596.2018.1482663

[20] Luna Z, Luker K. Reproductive justice. Annu Rev Law Soc Sci. 2013;9(1):327–52. doi: 10.1146/annurev-lawsocsci-102612-134037

[21] Hintz EA, Haywood A. Media frames of voluntary childlessness in the United States from 1989 to 2018. Sex Roles. 2020;84(11–12):747–64. doi: 10.1007/s11199-020-01197-z

[22] Giles D, Shaw RL, Morgan W. Representations of voluntary childlessness in the UK press, 1990—2008. J Health Psychol. 2009;14(8):1218–28. 19858341 10.1177/1359105309346341

[23] Peterson H. Absent non-fathers: gendered representations of voluntary childlessness in Swedish newspapers. Fem Media Stud. 2014;14(1):22–37.

[24] Fjell TI. Different voices on childfreedom (voluntary childfreeness) in the media and on the internet. Ethnol Scand. 2009;39:5–17.

[25] Graham M, Rich S. Representations of childless women in the Australian print media. Fem Media Stud. 2012;14(3):500–18. doi: 10.1080/14680777.2012.737346

[26] Rapoliene G, Sumskaite L. Depiction of childlessness in Lithuanian mass media from 2011–2016: a catalyst of modernization. RJCPR. 2019;21(3):19–36. doi: 10.21018/rjcpr.2019.3.280

[27] Stephani N. You still want to have kids, right? Representation of childfree women in Indonesian leading online news outlets. Media Asia. 2024;52(1):102–25. doi: 10.1080/01296612.2024.2335013

[28] Altheide DL, Schneider CJ. Qualitative media analysis. Sage Publications; 2012.

[29] Wallack LM. Media advocacy and public health: power for prevention. Sage; 1993.

[30] Hilton S, Hunt K. UK newspapers’ representations of the 2009–10 outbreak of swine flu: one health scare not over-hyped by the media? J Epidemiol Community Health. 2011;65(10):941–6. 21131303 10.1136/jech.2010.119875 PMC3171979

[31] Roslyng MM, Dindler C. Media power and politics in framing and discourse theory. Commun Theory. 2023;33(1):11–20.

[32] van Hulst M, Metze T, Dewulf A, de Vries J, van Bommel S, van Ostaijen M. Discourse, framing and narrative: three ways of doing critical, interpretive policy analysis. Crit Policy Stud. 2024;19(1):74–96. doi: 10.1080/19460171.2024.2326936

[33] Harcup T, O’neill D. What is news? Galtung and Ruge revisited. Jour Stud. 2001;2(2):261–80.

[34] Braun V, Clarke V. Conceptual and design thinking for thematic analysis. Qual Psychol. 2022;9(1):3–26. doi: 10.1037/qup0000196

[35] Braun V, Clarke V. Toward good practice in thematic analysis: avoiding common problems and be(com)ing a knowing researcher. Int J Transgend Health. 2022;24(1):1–6. doi: 10.1080/26895269.2022.2129597 36713144 PMC9879167

[36] Clarke V, Braun V. Thematic analysis. In: Encyclopedia of critical psychology. New York (NY): Springer; 2014. p. 1947–52. doi: 10.1007/978-1-4614-5583-7_311

[37] Ylänne V. Representations of ageing and infertility in the Twenty-First-Century British Press. In: The Palgrave handbook of infertility in history: approaches, contexts and perspectives. London: Palgrave Macmillan UK; 2017. p. 509–35.

[38] Burman E, Parker I. Discourse analytic research: repertoires and readings of texts in action. Routledge; 2016.

[39] Botelle R, Willott C. Birth, attitudes and placentophagy: a thematic discourse analysis of discussions on UK parenting forums. BMC Pregnancy Childbirth. 2020;20(1):134. doi: 10.1186/s12884-020-2824-3 32138706 PMC7059278

[40] De Simoni A, Shanks A, Mant J, Skelton JR. Making sense of patients’ internet forums: a systematic method using discourse analysis. Br J Gen Pract. 2014;64(620):e178-80. doi: 10.3399/bjgp14X677671 24567657 PMC3933853

[41] Yazdannik A, Yousefy A, Mohammadi S. Discourse analysis: a useful methodology for health-care system researches. J Educ Health Promot. 2017;6:111. doi: 10.4103/jehp.jehp_124_15 29296612 PMC5747223

[42] Evans-Agnew RA, Johnson S, Liu F, Boutain DM. Applying critical discourse analysis in health policy research: case studies in regional, organizational, and global health. Policy Polit Nurs Pract. 2016;17(3):136–46. doi: 10.1177/1527154416669355 27655739

[43] Solar O, Irwin A. A conceptual framework for action on the social determinants of health. WHO Document Production Services; 2010.

[44] de Souza R. The construction of HIV/AIDS in Indian newspapers: a frame analysis. Health Commun. 2007;21(3):257–66. doi: 10.1080/10410230701307733 17567257

[45] Kim S-H, Willis LA. Talking about obesity: news framing of who is responsible for causing and fixing the problem. J Health Commun. 2007;12(4):359–76. doi: 10.1080/10810730701326051 17558788

[46] Sieff E. Media frames of mental illnesses: the potential impact of negative frames. J Ment Health. 2003;12(3):259–69. doi: 10.1080/0963823031000118249

[47] Hart PS, Chinn S, Soroka S. Politicization and Polarization in COVID-19 News Coverage. Sci Commun. 2020;42(5):679–97. doi: 10.1177/1075547020950735 38602988 PMC7447862

[48] Zhang R. How media politicize COVID-19 lockdowns: a case study comparing frame use in the coverage of Wuhan and Italy lockdowns by The New York Times. Media Asia. 2021;48(2):89–107. doi: 10.1080/01296612.2021.1884518

[49] Carmines EG, Gerrity JC, Wagner MW. How abortion became a partisan issue: media coverage of the interest group‐political party connection. P&P. 2010;38(6):1135–58. doi: 10.1111/j.1747-1346.2010.00272.x

[50] Abbas AH. Politicizing COVID-19 vaccines in the press: a critical discourse analysis. Int J Semiot Law. 2022;35(3):1167–85. doi: 10.1007/s11196-021-09857-3 34276142 PMC8271332

[51] Ashburn-Nardo L. Parenthood as a moral imperative? Moral outrage and the stigmatization of voluntarily childfree women and men. Sex Roles. 2016;76(5–6):393–401. doi: 10.1007/s11199-016-0606-1

[52] Husnu S. The role of ambivalent sexism and religiosity in predicting attitudes toward childlessness in Muslim undergraduate students. Sex Roles. 2016;75(11–12):573–82. doi: 10.1007/s11199-016-0639-5

[53] van den Akker O, Fronek P, Blyth E, Frith L. ‘This neo-natal ménage à trois’: British media framing of transnational surrogacy. J Reprod Infant Psychol. 2015;34(1):15–27. doi: 10.1080/02646838.2015.1106454

[54] Tosam MJ, Chi PC, Munung NS, Oukem-Boyer OOM, Tangwa GB. Global health inequalities and the need for solidarity: a view from the Global South. Dev World Bioeth. 2018;18(3):241–9. doi: 10.1111/dewb.12182 29266755

[55] López-Bauta AA, Bustos C, Cova F. Subjective well-being of parents and childless adults in Chile. Curr Psychol. 2024;43(45):34828–40. doi: 10.1007/s12144-024-06946-x

[56] Alaazi DA, Ahola AN, Okeke-Ihejirika P, Yohani S, Vallianatos H, Salami B. Immigrants and the Western media: a critical discourse analysis of newspaper framings of African immigrant parenting in Canada. JEMS. 2021;47(19):4478–96.

[57] Varma A. Evoking empathy or enacting solidarity with marginalized communities? A case study of journalistic humanizing techniques in the San Francisco Homeless Project. Journ Stud. 2020;21(12):1705–23.

[58] Hadley RA. ‘No longer invincible’: the impact of involuntary childlessness on older men. Phys Ther Rev. 2021;26(5):328–43. doi: 10.1080/10833196.2021.1884172

[59] Amundsen D. A critical gerontological framing analysis of persistent ageism in NZ online news media: Don’t call us “elderly”! J Aging Stud. 2022;61:101009. doi: 10.1016/j.jaging.2022.101009 35654544

[60] Morley J, Cowls J, Taddeo M, Floridi L. Public health in the information age: recognizing the infosphere as a social determinant of health. J Med Internet Res. 2020;22(8):e19311. doi: 10.2196/19311 32648850 PMC7402642

[61] Palmer A, Gorman S. Misinformation, trust, and health: the case for information environment as a major independent social determinant of health. Soc Sci Med. 2025;381:118272. doi: 10.1016/j.socscimed.2025.118272 40513504

[62] Goulbourne T, Yanovitzky I. The communication infrastructure as a social determinant of health: implications for health policymaking and practice. Milbank Q. 2021;99(1):24. 33528043 10.1111/1468-0009.12496 PMC7984672

