# Supplementary material for: Framing reproductive narratives: A thematic discourse analysis of news representations of childlessness in 86 countries (2015–2025)
Source: PLOS Glob Public Health. 2026 Mar 11;6(3):e0005695. doi: 10.1371/journal.pgph.0005695 (PMC12978482; doi:10.1371/journal.pgph.0005695)
Supplement: S1 Table — (DOCX) [file pgph.0005695.s001.docx]

**S1 Table. List of news articles Included in the analysis.**

| **No** | **Year** | **Title** | **Media** | **Country** |
| --- | --- | --- | --- | --- |
| 1. | 2015 | The Brutal Truth About Being Childless at Work  <https://fortune.com/2015/11/07/truth-about-childless-at-work/> | Fortune | USA |
| 2. | 2015 | Single life, childless families rising in Iran  <https://www.tehrantimes.com/news/250257/Single-life-childless-families-rising-in-Iran> | Tehran Times | Iran |
| 3. | 2016 | Turkey’s Erdogan says childless women are ‘incomplete’  <https://www.aljazeera.com/news/2016/6/6/turkeys-erdogan-says-childless-women-are-incomplete> | Al Jazeera | Turkey |
| 4. | 2016 | End to long wait for the childless  <https://www.khaleejtimes.com/uae/end-to-long-wait-for-the-childless> | Khaleej Times | UAE |
| 5. | 2016 | Infertility - Stakeholders Seek Ways to Combat Stigmatisation Against African Women  <https://www.vanguardngr.com/2016/09/infertility-stakeholders-seek-ways-to-combat-stigmatisation-against-african-women/#google_vignette> | Vanguard | Nigeria |
| 6. | 2016 | I have been divorced from four marriages due to 'self-inflicted' infertility'  <https://www.mwananchi.co.tz/mw/habari/-nimeachika-kwenye-ndoa-nne-kisa-ugumba-wa-kujitakia--2798080> | Mwananchi | Tanzania |
| 7. | 2016 | Sturgeon: I revealed miscarriage to challenge assumptions about childless women leaders  <https://www.telegraph.co.uk/news/2016/09/04/nicola-sturgeon-i-disclosed-miscarriage-to-challenge-assumptions/> | The Telegraph | UK |
| 8. | 2016 | Alone and aging: Creating a safety net for isolated seniors  <https://eu.usatoday.com/story/news/2016/11/22/kaiser-alone-and-aging-creating-safety-net-isolated-seniors/94293674/> | USA Today Online | USA |
| 9. | 2016 | Inside story: Living childfree  <https://www.nzherald.co.nz/bay-of-plenty-times/news/inside-story-living-childfree/OVCH45V6XMZ2ZLF4XDWQQYHPJI/> | The New Zealand Herald | New Zealand |
| 10. | 2016 | The solitude of female politicians in South Asia  <https://www.aljazeera.com/opinions/2016/12/20/the-solitude-of-female-politicians-in-south-asia> | Al Jazeera | Multicountry |
| 11. | 2017 | Childless couples 'on track to be Australia's most common family type'  <https://www.abc.net.au/news/2017-05-15/childless-households-on-the-rise/8528546> | ABC | Australia |
| 12. | 2017 | Merck empowers Ugandan infertile women for the second year through 'Merck More than a Mother’ in partnership with Uganda Ministry of Health  <https://www.einpresswire.com/article/368489989/merck-empowers-ugandan-infertile-women-for-the-second-year-through-merck-more-than-a-mother-in-partnership-with-uganda-ministry-of-health> | Ein Presswire | Uganda |
| 13. | 2017 | The German trend towards childlessness has stopped  <https://www.welt.de/politik/deutschland/article167086499/Familien-Der-deutsche-Trend-zur-Kinderlosigkeit-ist-gestoppt.html> | Welt | Germany |
| 14. | 2017 | What's the secret to long life? Have children, Swedish experts say  <https://www.thelocal.se/20170314/people-with-kids-live-longer-swedish-study-shows>? | The Local | Sweden |
| 15. | 2017 | More Canadians living alone, with parents or without children: census  <https://www.nationalobserver.com/2017/08/02/news/more-canadians-living-alone-parents-or-without-children-census> | Canada’s National Observer | Canada |
| 16. | 2017 | Soaring childlessness among southern European women – report  <https://www.bbc.com/news/world-europe-38582100> | BBC | Multicountry |
| 17. | 2018 | Childlessness: Faith makes pregnant  <https://www.deutschlandfunk.de/kinderlosigkeit-glauben-macht-schwanger-100.html> | Deutschlandfunk | Italy |
| 18. | 2018 | Some Indian Couples Choose a Childfree Life  <https://www.theswaddle.com/childfree-in-india> | The Swaddle | India |
| 19. | 2018 | ‘Singles Tax’ Furor Highlights Sensitivity Over Pressure to Have More Children  <https://www.caixinglobal.com/2018-11-05/singles-tax-furor-highlights-sensitivity-over-pressure-to-have-more-children-101342216.html> | Caixing Global | China |
| 20. | 2018 | Childfree, happy: Women who shun motherhood  <https://nation.africa/kenya/life-and-style/dn2/childfree-happy-women-who-shun-motherhood-92982> | Nation Africa | Kenya |
| 21. | 2018 | Higher contributions for childless people – fair or discriminatory?  <https://www.zeit.de/politik/deutschland/2018-11/sozialbeitraege-jens-spahn-eltern-kinderlose-belastung-rechtslage-faq> | Zeit Online | Germany |
| 22. | 2019 | More than 1m childless people over 65 are 'dangerously unsupported'  <https://www.theguardian.com/science/2019/mar/28/over-1-million-childless-people-over-65-are-dangerously-unsupported> | The Guardian | UK |
| 23. | 2019 | Japan finance minister Aso sorry for criticizing childless  <https://apnews.com/general-news-f46fce9c0d3c449487fd71eaed8a5e1a> | Associated Press | Japan |
| 24. | 2019 | Childless women turn to WhatsApp for support  <https://www.heraldonline.co.zw/childless-women-turn-to-whatsapp-for-support/> | The Herald Zimbabwe | Zimbabwe |
| 25. | 2019 | First Lady seeks to empower childless couples  <https://www.heraldonline.co.zw/first-lady-seeks-to-empower-childless-couples/> | The Herald Zimbabwe | Ghana |
| 26. | 2019 | Childlessness has been increasing in SA – by women's choice  <https://www.news24.com/parent/family/parenting/childlessness-has-been-increasing-in-sa-by-womens-choice-20190322> | News 24 | South Africa |
| 27. | 2019 | The Arab world’s silent reproductive revolution  <https://www.aljazeera.com/news/2019/4/16/the-arab-worlds-silent-reproductive-revolution> | Al Jazeera | Qatar |
| 28. | 2019 | Radhia Al-Mutawakel, the "brave" Yemeni woman who does not have the courage to have a child  <https://www.bbc.com/arabic/middleeast-47991607> | BBC News Arabic | Yemen |
| 29. | 2019 | We are afraid of giving birth in this place now: Syrian women are afraid of motherhood  <https://www.bbc.com/arabic/middleeast-49085407> | BBC News Arabic | Syria |
| 30. | 2019 | One in six Czech 30-somethings likely to remain childless  <https://english.radio.cz/one-six-czech-30-somethings-likely-remain-childless-8136346> | Radio Prague International | Czech Republic |
| 31. | 2020 | Iran reins in family planning as population ages  <https://www.bbc.com/news/world-middle-east-53048719> | BBC | Iran |
| 32. | 2020 | No kids, no husband: the Filipino women defying society and embracing who they want to be  <https://www.scmp.com/lifestyle/family-relationships/article/3052263/no-kids-no-husband-filipino-women-defying-society> | South China Morning Post | Philippines |
| 33. | 2020 | Educated women are having fewer children — It’s not good for India’s demographic dividend  <https://theprint.in/opinion/educated-women-are-having-fewer-children-its-not-good-for-indias-demographic-dividend/1971404/> | The Print | India |
| 34. | 2020 | Waiting wombs: Rising above social stigma and demystifying childlessness  <https://nation.africa/kenya/news/waiting-wombs-rising-above-social-stigma-and-demystifying-childlessness-109794> | Nation Africa | Kenya |
| 35. | 2020 | Happy without children... it's possible!  <https://www.lefigaro.fr/sciences/heureux-sans-enfants-c-est-possible-20200113> | Le Figaro | France |
| 36. | 2020 | Why are fewer men becoming fathers than before?  <https://www.sciencenorway.no/children-and-adolescents-demography-gender-and-society/why-are-fewer-men-becoming-fathers-than-before/1767348> | Science Norway | Norway |
| 37. | 2020 | Parents Got More Time Off. Then the Backlash Started  <https://dj-factiva-com.eu1.proxy.openathens.net/article?id=drn:archive.newsarticle.NYTFEED020200905eg950018m> | The New York Times | USA |
| 38. | 2021 | Having children does not automatically result in a happier life  <https://www.oslomet.no/en/research/featured-research/having-children-does-not-automatically-result-in-happier-life> | Oslo Met | Norway |
| 39. | 2021 | War and Children: Why is a young woman urging her peers not to have children?  <https://www.bbc.com/arabic/middleeast-56351010> | BBC News Arabic | Syria |
| 40. | 2021 | Abstaining from childbearing: an individual right or a mutually agreed-upon decision?  <https://www.alaraby.com/news/%D8%A7%D9%84%D8%A7%D9%85%D8%AA%D9%86%D8%A7%D8%B9-%D8%B9%D9%86-%D8%A7%D9%84%D8%A5%D9%86%D8%AC%D8%A7%D8%A8-%D8%AD%D9%82-%D9%81%D8%B1%D8%AF%D9%8A-%D8%A3%D9%85-%D9%82%D8%B1%D8%A7%D8%B1-%D9%8A%D8%AA%D8%AE%D8%B0-%D8%A8%D8%B1%D8%B6%D9%89-%D8%A7%D9%84%D8%B7%D8%B1%D9%81%D9%8A%D9%86> | Al Araby | Yemen |
| 41. | 2021 | Child-free and happy: A group for Indonesians without kids – by choice  <https://www.thejakartapost.com/life/2021/04/14/child-free-and-happy-a-group-for-indonesians-without-kids-by-choice.html> | The Jakarta Post | Indonesia |
| 42. | 2021 | Defying a Culture: The Challenges of Being a Childfree Woman in Egypt  <https://egyptianstreets.com/2021/07/26/defying-a-culture-the-challenges-of-being-a-childfree-woman-in-egypt/> | Egyptian Streets | Egypt |
| 43. | 2021 | Why do some S'pore couples want to be child-free?  <https://www.straitstimes.com/singapore/community/pandemic-a-factor-behind-more-spore-couples-choosing-to-be-child-free> | The Straits Times | Singapore |
| 44. | 2021 | Pope expresses low birth alarm in Italy  <https://dj-factiva-com.eu1.proxy.openathens.net/article?id=drn:archive.newsarticle.NFINCE0020210514eh5e006hi> | CE Noticias Financieras | Italy |
| 45. | 2021 | China NPC: Three-child policy formally passed into law  <https://www.bbc.com/news/world-asia-china-58277473> | BBC | China |
| 46. | 2021 | Africa First Ladies Discuss Solutions to Infertility Stigma  <https://www.ktpress.rw/2021/04/africa-first-ladies-discuss-solutions-to-infertility-stigma/> | KT Press | Multicountry |
| 47. | 2022 | Latvian minister suggests adopting «childless family tax»  <https://bnn-news.com/latvian-minister-suggests-adopting-childless-family-tax-241344> | Baltic News Network | Latvia |
| 48. | 2022 | Having pets instead of kids robs us of ‘humanity’, pope says  <https://www.aljazeera.com/news/2022/1/5/having-pets-instead-of-kids-robs-us-of-humanity-pope-says> | Al Jazeera | Vatican City |
| 49. | 2022 | Marriage and Children: Who Would Accept a Partner Who Stipulates No Children?  <https://www.bbc.com/arabic/features-60047972> | BBC News Arabic | Multicountry |
| 50. | 2022 | Liberty, Equality & “Childfree” in France?  <https://frenchly.us/liberty-equality-maternity-childfree-in-france/> | Frenchly | France |
| 51. | 2022 | N. Korea to limit young, childless women from being dispatched to China for work  <https://www.dailynk.com/english/north-korea-limit-young-childless-women-being-dispatched-china-work/> | Daily NK | North Korea |
| 52. | 2023 | Childless in old age: “I’m not lonely!”  <https://www.srf.ch/kultur/gesellschaft-religion/kinderlos-im-alter-einsam-bin-ich-nicht-ueber-das-leben-im-alter-ohne-kinder> | Schweizer Radio und Fernsehen | Switzerland |
| 53. | 2023 | Report: Many married women do not want to increase the number of children  <https://www.mwananchi.co.tz/mw/habari/kitaifa/ripoti-wanawake-wengi-walioolewa-hawataki-kuongeza-watoto--4204680> | Mwananchi | Tanzania |
| 54. | 2023 | Vasectomy in Polish - Fatherhood? No, thank you  <https://dj-factiva-com.eu1.proxy.openathens.net/article?id=drn:archive.newsarticle.NWKEN00020230515ej5f0000g> | Newsweek | Poland |
| 55. | 2023 | Calls to not have children are on the rise in Egypt: challenges to norms and shocking survey results  <https://arabi21.com/story/1506026/%D8%AF%D8%B9%D9%88%D8%A7%D8%AA-%D8%B9%D8%AF%D9%85-%D8%A7%D9%84%D8%A5%D9%86%D8%AC%D8%A7%D8%A8-%D8%AA%D8%AA%D8%B2%D8%A7%D9%8A%D8%AF-%D8%A8%D9%85%D8%B5%D8%B1-%D8%AA%D8%AD%D8%AF%D9%8A%D8%A7%D8%AA-%D9%84%D9%84%D8%A3%D8%B9%D8%B1%D8%A7%D9%81-%D9%88%D9%86%D8%AA%D8%A7%D8%A6%D8%AC-%D9%85%D8%B3%D8%AD-%D8%B5%D8%A7%D8%AF%D9%85%D8%A9> | Arabi21 | Egypt |
| 56. | 2023 | 'Devalued product': China mother plunges into depression over feeling inferior about unmarried 30-something daughter, sparks online debate  <https://www.scmp.com/news/people-culture/trending-china/article/3242203/devalued-product-china-mother-plunges-depression-over-feeling-inferior-about-unmarried-30-something> | South China Morning Post | China |
| 57. | 2023 | More Vietnamese marriages going childless  <https://e.vnexpress.net/news/trend/more-vietnamese-marriages-going-childless-4603904.html> | VN Express | Vietnam |
| 58. | 2023 | Childfree Phenomenon in Indonesia Not Yet Worrying According to BKKBN  <https://www.tempo.co/gaya-hidup/fenomena-childfree-di-indonesia-belum-mengkhawatirkan-menurut-bkkbn-220171> | Tempo | Indonesia |
| 59. | 2023 | The world's most powerful men want women to have more babies  <https://www.businessinsider.com/powerful-men-women-more-babies-xi-putin-kim-jong-un-2023-12> | Business Insider | Multicountry |
| 60. | 2023 | “I can’t support a child until I’m 70” Even men in their 30s are choosing DINKs.  <https://n.news.naver.com/mnews/article/025/0003285472?sid=101> | Joongang Ilbo | South Korea |
| 61. | 2023 | With the low birth rate stuck and no solution found: 21% of those in their 20s to 50s “in favor of single tax”  <https://n.news.naver.com/mnews/article/023/0003773040?sid=102> | Chosun Ilbo | South Korea |
| 62. | 2023 | The North Korean leader calls for women to have more children to halt a fall in the birthrate  <https://apnews.com/article/north-korea-kim-jong-un-birthrate-72d59d0e2c685549ba8cd8734d8a7fe2> | Associated Press | North Korea |
| 63. | 2023 | Challenges of old age without children  <https://mujeres.expansion.mx/opinion/2023/08/29/desafios-de-una-vejez-sin-hijos-as> | Expansion Mujeres | Mexico |
| 64. | 2024 | Half of Mexican women do not want to have children  <https://www.jornada.com.mx/noticia/2024/07/26/opinion/la-mitad-de-las-mexicanas-no-desean-tener-hijos-6235> | La Jornada | Mexico |
| 65. | 2024 | Not having children: a private decision with social consequences  <https://www.eltiempo.com/mundo/latinoamerica/no-tener-hijos-una-decision-privada-con-consecuencias-sociales-3407107> | El Tiempo | Multicountry |
| 66. | 2024 | Pope praises Indonesians for choosing children over cats  <https://www.reuters.com/world/asia-pacific/pope-praises-indonesians-choosing-children-over-cats-2024-09-04/> | Reuters | Indonesia |
| 67. | 2024 | BKKBN: KIA Law protects Indonesia from “childfree” phenomenon  <https://www.antaranews.com/berita/4194855/bkkbn-uu-kia-lindungi-indonesia-dari-fenomena-childfree> | Antara | Indonesia |
| 68. | 2024 | We Finally Decided Not to Have Children  <https://www.kompas.id/artikel/akhirnya-kami-memutuskan-tidak-punya-anak-7> | Kompas | Indonesia |
| 69. | 2024 | Malaysian minister says 'Childfree' trend contradicts Islamic teachings  <https://www.nationthailand.com/news/world/40039244> | Nation Thailand | Malaysia |
| 70. | 2024 | Worrying about your child’s future is not a reason to refuse to have children  <https://www.pressreader.com/malaysia/berita-harian-malaysia/20240709/281732684699212> | Berita Harian Malaysia | Malaysia |
| 71. | 2024 | Thailand is one of the world’s fastest-ageing developing nations. This is how it happened  <https://www.channelnewsasia.com/cna-insider/thailand-fast-ageing-population-declining-birth-rate-children-expensive-4406151> | Channel NewsAsia | Thailand |
| 72. | 2024 | Choosing to be child-free in an ‘apocalyptic’ South Asia  <https://www.aljazeera.com/features/2024/10/13/choosing-to-be-child-free-in-an-apocalyptic-south-asia> | Al Jazeera | Pakistan |
| 73. | 2024 | Couples who remain childless by choice face societal othering  <https://kathmandupost.com/national/2024/05/25/couples-who-remain-childless-by-choice-face-societal-othering> | Kathmandu Post | Nepal |
| 74. | 2024 | Why South Korean women aren't having babies  <https://www.bbc.com/news/world-asia-68402139> | BBC | South Korea |
| 75. | 2024 | Half of adults say “it’s okay not to have children”  <https://n.news.naver.com/mnews/article/028/0002722882?sid=102> | Hangyeoreh | South Korea |
| 76. | 2024 | Ultra-low birth rate, the shadow of a life without leisure  <https://www.hani.co.kr/arti/opinion/column/1148449.html> | Hangyeoreh | South Korea |
| 77. | 2024 | Let's create a world we want to show our children, a society where children are happy  <https://n.news.naver.com/mnews/article/023/0003866100?sid=004> | Chosun Ilbo | South Korea |
| 78. | 2024 | Birth rate and female employment must be stable to increase  <https://n.news.naver.com/mnews/article/032/0003272163?sid=110> | Kyunghyang Shinmoon | South Korea |
| 79. | 2024 | Birth rate collapses to 0.7: If this continues, ‘extinction due to population decline’ will become a reality  <https://www.donga.com/news/Opinion/article/all/20240228/123745741/>1 | Donga Ilbo | South Korea |
| 80. | 2024 | Investigation: Why aren’t North Korea’s women having babies anymore? (1) — The fertility rate is already severely low. It’s rare to see anyone carrying babies around  <https://www.asiapress.org/rimjin-gang/2024/04/society-economy/nobaby1/> | Asia Press | North Korea |
| 81. | 2024 | Investigation: Why aren’t North Korea’s women having babies anymore? (2) — Women increasingly avoid marriage to protect themselves. Men are considered a burden to women in N. Korean society  <https://www.asiapress.org/rimjin-gang/2024/04/society-economy/nobaby2/> | Asia Press | North Korea |
| 82. | 2024 | Investigation: Why aren’t North Korea’s women having babies anymore? (3) — 'We aren't stupid, we don't want have kids who’ll turn into homeless'  <https://www.asiapress.org/rimjin-gang/2024/04/recommendations/nobaby3/> | Asia Press | North Korea |
| 83. | 2024 | Seniors looking after one another  <https://www.chinadaily.com.cn/a/202402/21/WS65d549b9a31082fc043b8374.html> | China Daily | China |
| 84. | 2024 | 73% of U-35 women in Finland are childless  <https://www.dailyfinland.fi/health/35840/73-of-U-35-women-in-Finland-are-childless> | Daily Finland | Finland |
| 85. | 2024 | Only 2.4% of women in Russia want to be childfree  <https://www.forbes.ru/forbes-woman/523826-cajldfri-hotat-byt-tol-ko-2-4-zensin-v-rossii> | Forbes Russia | Russia |
| 86. | 2024 | The State Duma is talking about a tax for childless Russians  <https://news.ru/vlast/v-gosdume-predlozhili-zastavit-rossiyan-platit-za-bezdetnost> | News.ru | Russia |
| 87. | 2024 | Kremlin finds new enemies to target — Russians without kids  <https://kyivindependent.com/kremlin-finds-new-enemy-to-target-russians-without-kids/> | The Kyiv Independent | Russia |
| 88. | 2024 | As Russia targets abortion and ‘childfree propaganda’ to raise birth rates, ‘Pregnant at 16’ reality show rebrands to make motherhood more appealing  <https://meduza.io/en/feature/2024/11/05/as-russia-targets-abortion-and-childfree-propaganda-to-raise-birth-rates-pregnant-at-16-reality-show-rebrands-to-make-motherhood-more-appealing> | Meduza | Russia |
| 89. | 2024 | Russia is shrinking; the Kremlin says child-free ideology is to blame  <https://www.washingtonpost.com/world/2024/11/10/russia-population-demography-children-family/> | Washington Post | Russia |
| 90. | 2024 | Harris says it's 'not the 1950s anymore' in dismissing criticism over not having biological children  <https://www.wral.com/story/harris-says-it-s-not-the-1950s-anymore-in-dismissing-criticism-over-not-having-biological-children/21660699/> | CNN | USA |
| 91. | 2024 | Child-free Republican candidate borrows wife and children from friend to pose for campaign photos  <https://www.independent.co.uk/news/world/americas/us-politics/derrick-anderson-photo-virginia-b2621414.html> | Independent Online | USA |
| 92. | 2024 | The friends who came together to build a villa to live in together in old age  <https://dj-factiva-com.eu1.proxy.openathens.net/article?id=drn:archive.newsarticle.NFINCE0020240410ek4a00cjj> | CE Noticias Financieras | Brazil |
| 93. | 2024 | Does declining birth rates pose a threat to Arab countries?  <https://www.aljazeera.net/family/2024/12/16/%D9%87%D9%84-%D9%8A%D8%B4%D9%83%D9%84-%D8%AA%D8%B1%D8%A7%D8%AC%D8%B9-%D9%85%D8%B9%D8%AF%D9%84%D8%A7%D8%AA-%D8%A7%D9%84%D8%A5%D9%86%D8%AC%D8%A7%D8%A8-%D8%AE%D8%B7%D8%B1%D8%A7-%D8%B9%D9%84%D9%89> | Al Jazeera | Multicountry |
| 94. | 2024 | Women in Argentina are giving birth later and less, report shows  <https://www.batimes.com.ar/news/culture/women-in-argentina-are-giving-birth-later-and-less-report-shows.phtml> | Buenos Aires Times | Argentina |
| 95. | 2024 | Childless by Choice, A Private Decision with Social Consequences  <https://www.connectas.org/childless-by-choice-a-private-decision-with-social-consequences/> | Connectas | Chile |
| 96. | 2024 | Fertility centre offers glimmer of hope to childless couples  <https://timesofoman.com/article/148770-fertility-centre-offers-glimmer-of-hope-to-childless-couples> | Times of Oman | Oman |
| 97. | 2024 | Aging and declining fertility rates: Morocco faces economic and societal challenges  <https://www.hespress.com/%D8%A7%D9%84%D8%B4%D9%8A%D8%AE%D9%88%D8%AE%D8%A9-%D9%88%D8%AA%D8%B1%D8%A7%D8%AC%D8%B9-%D9%85%D8%B9%D8%AF%D9%84-%D8%A7%D9%84%D8%AE%D8%B5%D9%88%D8%A8%D8%A9-%D8%A7%D9%84%D9%85%D8%BA%D8%B1%D8%A8-%D8%A3-1483686.html> | Hespress | Morocco |
| 98. | 2024 | Increasing aging and declining fertility: An expert warns of serious implications for the sustainability of the national economy  <https://www.thevoice.ma/%D8%AA%D8%B2%D8%A7%D9%8A%D8%AF-%D8%A7%D9%84%D8%B4%D9%8A%D8%AE%D9%88%D8%AE%D8%A9-%D9%88%D8%A7%D9%86%D8%AE%D9%81%D8%A7%D8%B6-%D8%A7%D9%84%D8%AE%D8%B5%D9%88%D8%A8%D8%A9-%D8%AE%D8%A8%D9%8A%D8%B1-%D9%8A/> | The Voice | Morocco |
| 99 | 2024 | The "European curse" also reaches Morocco: its population is aging, but more slowly  <https://dj-factiva-com.eu1.proxy.openathens.net/article?id=drn:archive.newsarticle.NFINCE0020241115ekbf003gf> | CE Noticias Financieras | Morocco |
| 100. | 2024 | Heba Auf: Marriage is not an obligation...and not having children is permissible in this case.  <https://www.masrawy.com/islameyat/others-islamic_ppl_news/details/2024/9/8/2639755/%D9%87%D8%A8%D8%A9-%D8%B9%D9%88%D9%81-%D8%A7%D9%84%D8%B2%D9%88%D8%A7%D8%AC-%D9%84%D9%8A%D8%B3-%D9%81%D8%B1%D8%B6%D8%A7-%D9%88%D8%B9%D8%AF%D9%85-%D8%A7%D9%84%D8%A7%D9%86%D8%AC%D8%A7%D8%A8-%D8%AD%D9%84%D8%A7%D9%84-%D9%81%D9%89-%D9%87%D8%B0%D9%87-%D8%A7%D9%84%D8%AD%D8%A7%D9%84%D8%A9-> | Masrawy | Egypt |
| 101. | 2024 | The real reason for the rise in male childlessness  <https://www.bbc.com/news/articles/cp81ynn7r4mo> | BBC | UK |
| 102. | 2024 | The crushing truth about being childless at 64  <https://www.telegraph.co.uk/health-fitness/parenting/children/childless/> | The Telegraph | UK |
| 103. | 2024 | Families are shrinking, thinning out, and becoming more vertical  <https://english.elpais.com/society/2024-01-14/families-are-shrinking-thinning-out-and-becoming-more-vertical.html> | El Pais | Spain |
| 104. | 2024 | The 'I want to, but I can't' of having children: in search of the obstacles that hinder birth rates in Spain  <https://www.eleconomista.es/economia/noticias/13024439/10/24/el-quiero-y-no-puedo-de-tener-hijos-en-busca-de-las-trabas-que-frenan-la-natalidad-en-espana.html> | El Economista | Spain |
| 105. | 2024 | 77% of Spaniards believe that they do not have more children due to a lack of financial means  <https://www.rtve.es/noticias/20241008/mas-mitad-personas-sin-hijos-ser-padres-segun-cis/16278674.shtml> | RTVE | Spain |
| 106. | 2024 | "I live in a rental and I don't have a stable life, how am I going to start a family?": not having children in Spain, a personal choice and a life-forced choice in equal measure.  <https://cadenaser.com/nacional/2024/11/24/vivo-de-alquiler-y-no-tengo-una-vida-estable-como-voy-a-formar-una-familia-no-tener-hijos-en-espana-una-eleccion-personal-y-obligada-por-la-vida-a-partes-iguales-cadena-ser/> | Cadenaser | Spain |
| 107. | 2024 | Situation in the world, Netherlands a big reason behind decision not to have children  <https://nltimes.nl/2024/11/27/situation-world-netherlands-big-reason-behind-decision-children> | NL Times | Netherlands |
| 108. | 2024 | Late Pain: “I am the last of my family, they will perish with me.”  <https://www.sueddeutsche.de/leben/maenner-kinderlosigkeit-kinderwunsch-lux.F2ZFEF8knWr9RaMKV8tKHb?reduced=true> | Süddeutsche Zeitung | Germany |
| 109. | 2024 | The new agreement will help the involuntarily childless to become pregnant  <https://dj-factiva-com.eu1.proxy.openathens.net/article?id=drn:archive.newsarticle.ATPHAM0020240217ek2e000ke> | Contify Life Science News | Denmark |
| 110. | 2024 | Leader: Population crisis - or just an effect of more freedom?  <https://app.retriever-info.com/go-article/0509152024012995023ca119c464a4e6db5b1a9583d4b9/null/archive/search?type=jwt> | Dagens Nyheter | Sweden |
| 111. | 2024 | Having children is not a human right  <https://app.retriever-info.com/go-article/057349202406148fda41c6d3ed2a7ed490b6d891c3d864/null/archive/search?type=jwt> | Aftonbladet | Sweden |
| 112. | 2024 | Women have the right to their own bodies  <https://app.retriever-info.com/go-article/05080320240618eaa1e64c0d70da25d1aacdba32e42f92/null/archive/search?type=jwt> | Svenska Dagbladet | Sweden |
| 113. | 2024 | "It's not a lack of money - hopelessness is behind childlessness"  <https://app.retriever-info.com/go-article/0508032024082977a509ddf1faf9f21337ee84f712aaed/null/archive/search?type=jwt> | Svenska Dagbladet | Sweden |
| 114. | 2024 | Older adults without children bear lion’s share of caregiving for parents: S’pore study  <https://www.straitstimes.com/singapore/older-adults-without-children-bear-lion-s-share-of-caregiving-for-parents-s-pore-study> | The Straits Times | Singapore |
| 115. | 2024 | Number of lone, childless, old men likely to hit 5.2 million in ’50  <https://www.asahi.com/ajw/articles/15561893> | The Asahi Shimbun | Japan |
| 116. | 2024 | Why So Many Hungarians Are Staying Childfree  <https://www.rferl.org/a/children-hungary-child-free-movement/33146567.html> | Radio Free Europe Radio Liberty | Hungary |
| 117. | 2025 | "Contemporary times are wrong - a defense of parenthood"  <https://app.retriever-info.com/go-article/05091520250102d22e307a798cbab58939d37aec6d8a49/null/archive/search?type=jwt> | Dagens Nyheter | Sweden |
| 118. | 2025 | After the adoption collapse - the surrogacy boom is here: Billion-dollar industry on the rise globally • Illegal in Swedish healthcare  <https://app.retriever-info.com/go-article/05712520250209e8df484e105cf46b70d9ea33c51471eb/null/archive/search?type=jwt> | DagensETC | Sweden |
| 119. | 2025 | This is why Swedish women don't have children anymore  <https://app.retriever-info.com/go-article/05747620250331829b269f3c7b74014ce43904806f70ce/null/archive/search?type=jwt> | Skaraborgs Läns Tidning | Sweden |
| 120. | 2025 | One in five women in Germany is childless  <https://www.brigitte.de/leben/kinderlosenquote--wieso-jede-fuenfte-frau-in-deutschland-keine-kinder-will--13543916.html> | Brigitte | Germany |
| 121. | 2025 | Fatwa Secretary: Not having children is permissible according to Islamic law, and planning does not conflict with religion  <https://news.twaslnews.com/%D8%A7%D9%84%D8%AC%D9%85%D9%87%D9%88%D8%B1/54744/> | Tawasol News | Egypt |
| 122. | 2025 | Declining fertility rate causes concern in UAE  <https://dj-factiva-com.eu1.proxy.openathens.net/article?id=drn:archive.newsarticle.GLFNWS0020250131el1v0000c> | Gulf News | UAE |
| 123. | 2025 | The spectre of aging may threaten our society  <https://www.alwatan.com.sa/article/1163024> | Alwatan | Saudi Arabia |
| 124. | 2025 | Latifa: I am happy with my decision not to have children and I have no regrets  <https://syria.news/f6450288-18022512.html> | Syria News | Tunisia |
| 125. | 2025 | Why young, childless men are having vasectomies  <https://dj-factiva-com.eu1.proxy.openathens.net/article?id=drn:archive.newsarticle.NFINCE0020250318el3i005b8> | CE Noticias Financieras | Brazil |
| 126. | 2025 | Deciding not to have children: a growing choice with cultural and social factors at play  <https://www.lavoz.com.ar/ciudadanos/decidir-no-tener-hijos-una-eleccion-en-crecimiento-con-factores-culturales-y-sociales-en-juego/> | Lavoz | Argentina |
| 127. | 2025 | In Chile, having children is a thing for the brave  <https://elpais.com/chile/2025-04-16/en-chile-tener-hijos-es-cosa-de-valientes.html> | El Pais | Chile |
| 128. | 2025 | First childless generation reaches old age: the outlook they face  <https://www.portafolio.co/tendencias/sociales/primera-generacion-sin-hijos-llega-a-la-vejez-el-panorama-al-que-se-enfrenta-627922> | Portafolio | Colombia |
| 129. | 2025 | Kazakhstan may introduce a childlessness tax. What the Ministry of Finance says  <https://en.tengrinews.kz/kazakhstan_news/kazakhstan-may-introduce-a-childlessness-tax-what-the-266556/> | Tengri News | Kazakhstan |
| 130. | 2025 | Latvia does not lack bureaucrats, but catastrophically lacks young mothers, says MP  <https://bnn-news.com/latvia-does-not-lack-bureaucrats-but-catastrophically-lacks-young-mothers-says-mp-265951> | Baltic News Network | Latvia |
| 131. | 2025 | Beyond the ‘Divine Blessing’: How Medhin Hagos’ Late Motherhood Exposes Society’s Double Standards and Demands a Shift from Scrutiny to Structural Reflection  <https://borkena.com/2025/03/04/late-motherhood-beyond-the-divine-blessing-how-medhin-hagos-late-motherhood/> | Borkena | Ethiopia |
