# Supplementary material for: Framing reproductive narratives: A thematic discourse analysis of news representations of childlessness in 86 countries (2015–2025)
Source: PLOS Glob Public Health. 2026 Mar 11;6(3):e0005695. doi: 10.1371/journal.pgph.0005695 (PMC12978482; doi:10.1371/journal.pgph.0005695)
Supplement: S2 Table — (DOCX) [file pgph.0005695.s002.docx]

**S2 Table. Illustrative audit trail documenting the thematic analysis process.**

| **Quotes from the News Article** | **Initial Codes** | **Refined Codes** | **Theme** |
| --- | --- | --- | --- |
| *On November 28, Russian leader Vladimir Putin urged Russian women to return to their roots and, like their "grandmothers and great-grandmothers," have "seven, eight, or even more children."*  *"Let us preserve and revive these excellent traditions. Large families must become the norm, a way of life for all of Russia's people," Putin said at the World Russian People's Council in Moscow.*  *"Preserving and increasing the population of Russia is our goal for the coming decades and even generations ahead. This is the future of the Russian world, the millennium-old, eternal Russia," he added.* | Putin urged Russian women  return to their roots  like grandmothers and great-grandmothers  have seven, eight, or even more children  preserve and revive excellent traditions  large families must become the norm  a way of life for all Russians  preserving and increasing population  our goal for the coming decades  even generations ahead  the future of Russian world  the millenium-old eternal Russia | state intervention to private lives  traditional women’s role  women should give birth to more babies  women under pressure  women’s national responsibility  maintaining country’s population  large families as the norm  Russian traditional norms  Russian population crisis  population policy | The Guinea Pig of the State |
| *Pope Francis has risked the ire of the world’s childless dog and cat owners, suggesting people who substitute pets for kids exhibit “a certain selfishness”. Speaking on parenthood during a general audience on Wednesday at the Vatican, Francis lamented that pets “sometimes take the place of children” in society. The practice, said the head of the world’s 1.3 billion Catholics, “is a denial of fatherhood and motherhood and diminishes us, takes away our humanity”. Thus, “civilisation grows old without humanity because we lose the richness of fatherhood and motherhood, and it is the country that suffers”, the pontiff said at the Paul VI Hall. In 2014, the 85-year-old told Il Messaggero daily that having pets instead of children was “another phenomenon of cultural degradation”, and that emotional relationships with pets was “easier” than the “complex” relationship between parents and children.* | childless dog and cat owners  people who substitute pets for kids  exhibit a certain selfishness  pets take place of children in society  the practice denial of fatherhood and motherhood  diminishes us  takes away our humanity  civilization grows old without humanity  lose the richness of fatherhood and motherhood  the country that suffers  another phenomenon of cultural degradation  emotional relationship with pets was easier  than complex relationship between parents and children | childless animal lovers  animal replace children  selfish childless people  pets replace kids in society  denial of parenthood  threat to humanity  impact to country population  cultural degradation phenomenon  childless choose easier way  pets require less complex relationship  children need more efforts than pets | Crazy Rich Selfish Animal Lovers |
| *“We decided not to have kids right away. I mean, we were still a bit confused, but we decided when I was 33-34 that we will not have kids. We were both in love with our work. We’re very focused on ensuring that we give our bit back to society. My husband was totally devoted to an organisation and didn’t want any liabilities. I worked on a lot of pro bono cases, and my field is pretty demanding. We just decided that we’d rather do what we’re doing well, than be greedy and want more, and end up being unhappy and unfulfilled. We’re quite happy with our decision. We’ve been very busy and very productive.* | decided not to have kids right away  were still a bit confused  decided at 33-34 not to have kids  both in love with our work  very focused ensuring give back to society  husband devoted to an organisation  husband didn’t want any liabilities  worked a lot of pro bono cases  my field is pretty demanding  rather do what we’re doing well  than be greedy and want more  end up unhappy and unfulfilled  quite happy with our decision  very busy and very productive | conscious decision being childless  decided to be childless at 30s  choosing career over children  contribute to society through work  partner also refuse fatherhood  couple happy with their decision | No Baby, No Cry |
| *“I often urge my young friends, both men and women, not to marry, and if they do so for love, I tell them to think carefully before they commit themselves to having a child and putting him or her into this life, because he or she will be thrown into the jaws of war and hunger, just as we were. I decided not to marry, no matter how much pressure my family and society put on me, because I don't want to commit a crime against a child and throw him into the misery we're suffering now... I've seen enough orphans, hungry children, sick children, and dead children.”* | often urge my young friends  not to marry  if doing it for love think carefully  before committing having a child  putting children into this life  will be thrown into the jaws of hunger and war  just as we were  decided not to marry  no matter how much pressure  from my family and society put on me  don’t want to commit a crime against a child  throw him into the misery  we’re suffering now  seen enough orphans, hungry children, sick children, dead children | young people avoid marriage  love versus logic  a child as commitment  bring children into life  a life with hunger and war  choose not to marry  pressure from family and society  a crime against a child  put the same suffering into a child  unlucky and unhappy children | Bringing Children Into a Broken World |
| *That’s another layer of isolation as I age, and so is the fact that now, in my 60s, some of my friends are becoming grandfathers. For a brief few years they’d had the time to come out for lunch on the weekends, and while I’m over the moon for them and their families, it’s hard to see that companionship slowly disappear again. That’s not to say I don’t have a good life. As well as having a brilliant partner, my own experiences with childlessness have led me to a new career in researching the impacts of infertility and childlessness on men.* | another layer of isolation  as I age  the fact now  in my 60s  some friends becoming grandfathers  they’d had time to come out  lunch on the weekends  over the moon for them and their families  hard to see that companionship slowly disappear again  not to say don’t have a good life  having a brilliant partner  own experience with childlessness  led me to a new career  researching impacts of fertility and childlessness on men | layer of isolation during ageing  childless older men  comparing life with friends  over the moon with friends’ families  longing for family companionship  self-reflecting about own life | Winter Regret and Loneliness |
